# Supplementary material for: AcuB senses cellular energy charge to coordinate acetyl-CoA synthesis in bacteria
Source: Nat Commun. 2026 Apr 24;17:3815. doi: 10.1038/s41467-026-71006-w (PMC13121692; doi:10.1038/s41467-026-71006-w)
Supplement: Supplementary file 1 — Supplementary Information [file 41467_2026_71006_MOESM1_ESM.pdf]

# SUPPLEMENTARY INFORMATION

Markus Janetzky[1], Norman Geist[2], Sabrina Schulze[1], Henriette Rückert[1], Kimani Gatzemeyer[1], Gottfried J. Palm[1], Leona Berndt[1], Britta Girbardt[1], Daniel Weis[1], Ina Menyes[3], Norma Welsch[4], Thomas Schweder[4], Mark Dörr[3], Stefan Kemnitz[5], Uwe T. Bornscheuer[3], Mihaela Delcea[2] and Michael Lammers[1*]

[1]University of Greifswald, Institute of Biochemistry, Department of Synthetic and Structural Biochemistry, 17489 Greifswald, Germany
[2]University of Greifswald, Institute of Biochemistry, Department of Biophysical Chemistry, 17489 Greifswald, Germany
[3]University of Greifswald, Institute of Biochemistry, Department of Biotechnology & Enzyme Catalysis, 17489 Greifswald, Germany
[4]University of Greifswald, Institute of Pharmacy, Department of Pharmaceutical Biotechnology, 17489 Greifswald, Germany
[5]University of Greifswald, University Computing Center, Department for High Performance Computing, 17489 Greifswald, Germany

[*]correspondence should be addressed to Michael Lammers; Email: michael.lammers@uni-greifswald.de; Tel.: 03834-420-4356; Fax: 03834-420-4373

Supplementary Figures 1-35
Supplementary Tables 1-10
Uncropped images of Supplementary Figures

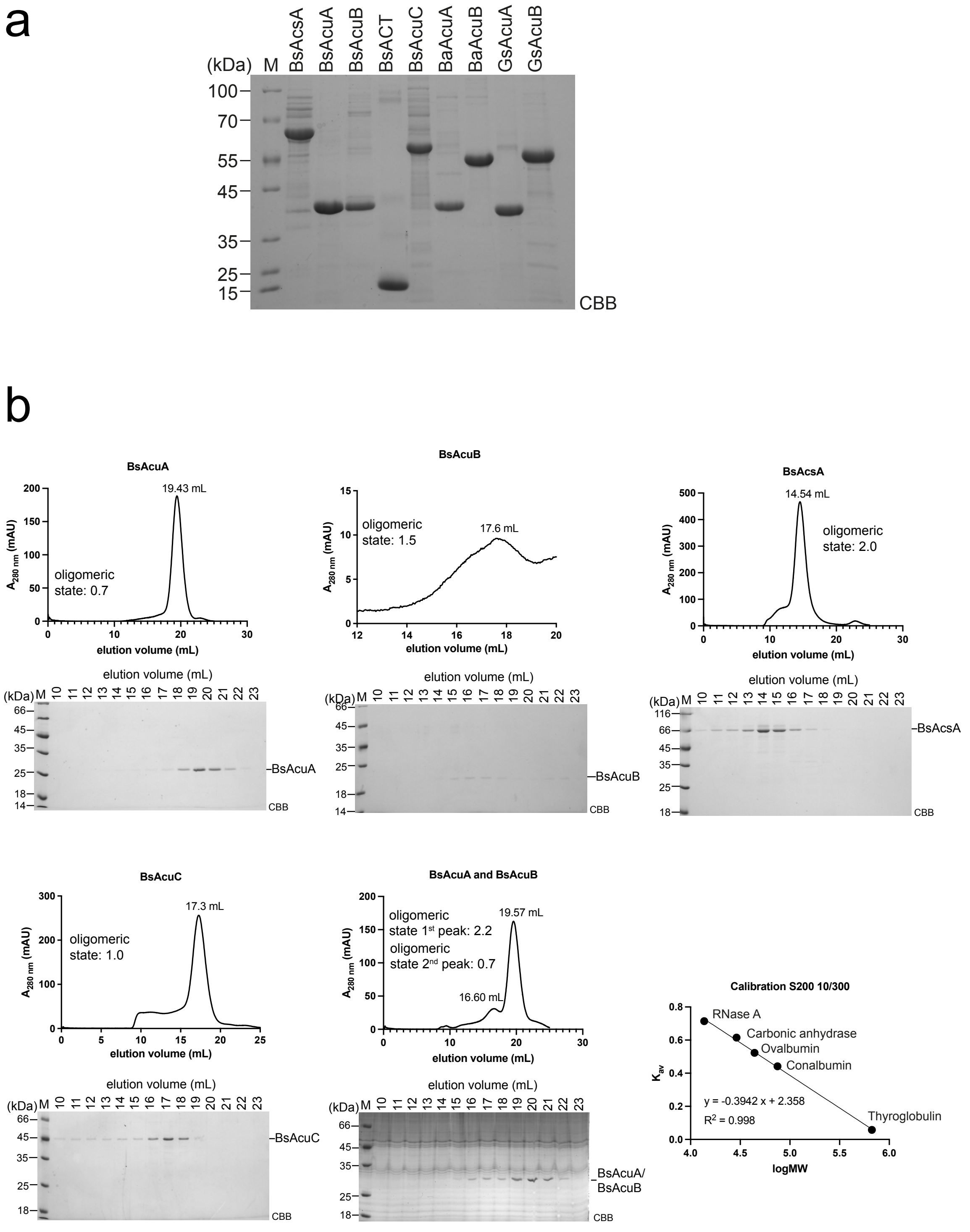


**Supplementary Figure 1: Final purity of proteins expressed and purified in this study and analytical size-exclusion chromatography (SEC) of the *B. subtilis* proteins.**

**a.** Final purity of the *B. subtilis* proteins BsAcsA, BsAcuA, BsAcuB, BsACT, BsAcuC (Bs: *B. subtilis*), of the *B. amyloliquefaciens* proteins BaAcuA, BaAcuB (Ba: *B. amyloliquefaciens*) and the *G. stearothermophilus* proteins GsAcuA and GsAcuB purified in this study (Gs: *G. stearothermophilus*). The lane labelled with M represents the protein molecular weight marker. The SDS-PAGE gel was stained with Coomassie-brilliant blue (CBB). Source data are provided as Source Data File.

**b.** Analytical SEC was conducted to determine the apparent molecular weights of the *B. subtilis* proteins BsAcsA, BsAcuA, BsAcuB, BsACT, BsAcuC. Moreover, BsAcuA and BsAcuB were evaluated together indicating both do not form a stable complex on analytical SEC. Elution was followed by recording the absorption at 280 nm ($A_{280\ nm}$) in milli absorbance units (mAU). Source data are provided as Source Data file.

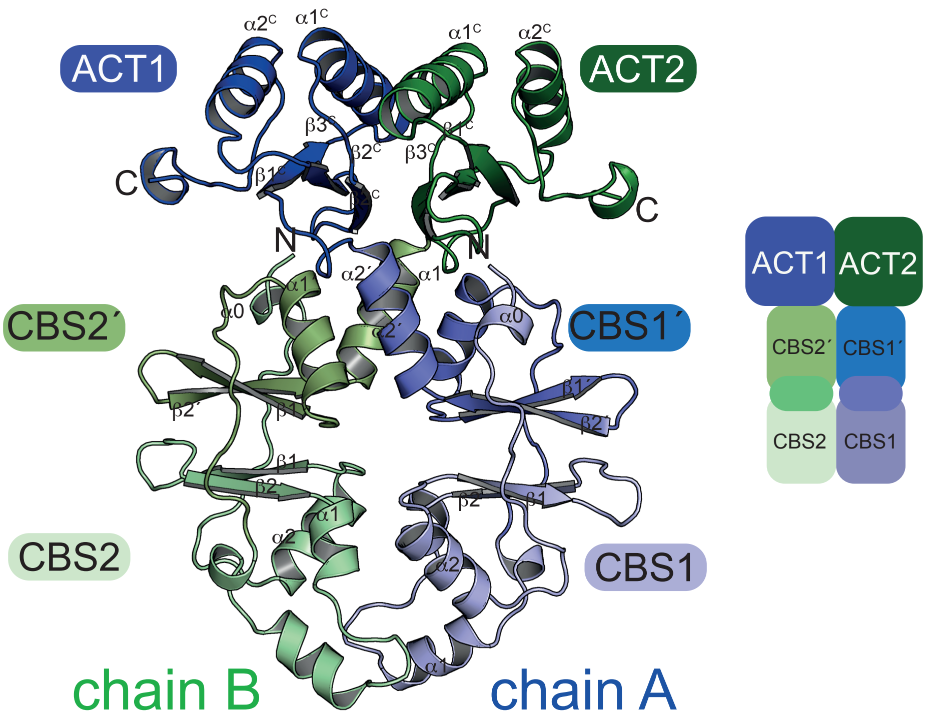


**Supplementary Figure 2: AlphaFold3 structure prediction of *Bacillus subtilis* AcuB, BsAcuB.** The AlphaFold3 structure prediction suggest AcuB forming a dimer with each monomer encompassing of an N-terminal Bateman domain, each of which is composed of two CBD-modules, and a C-terminal ACT domain. The AlphaFold3 confidence score (ipTM+pTM=0.69+0.70) suggests an overall correct fold of the complex. The figure was created with PyMOL[1].

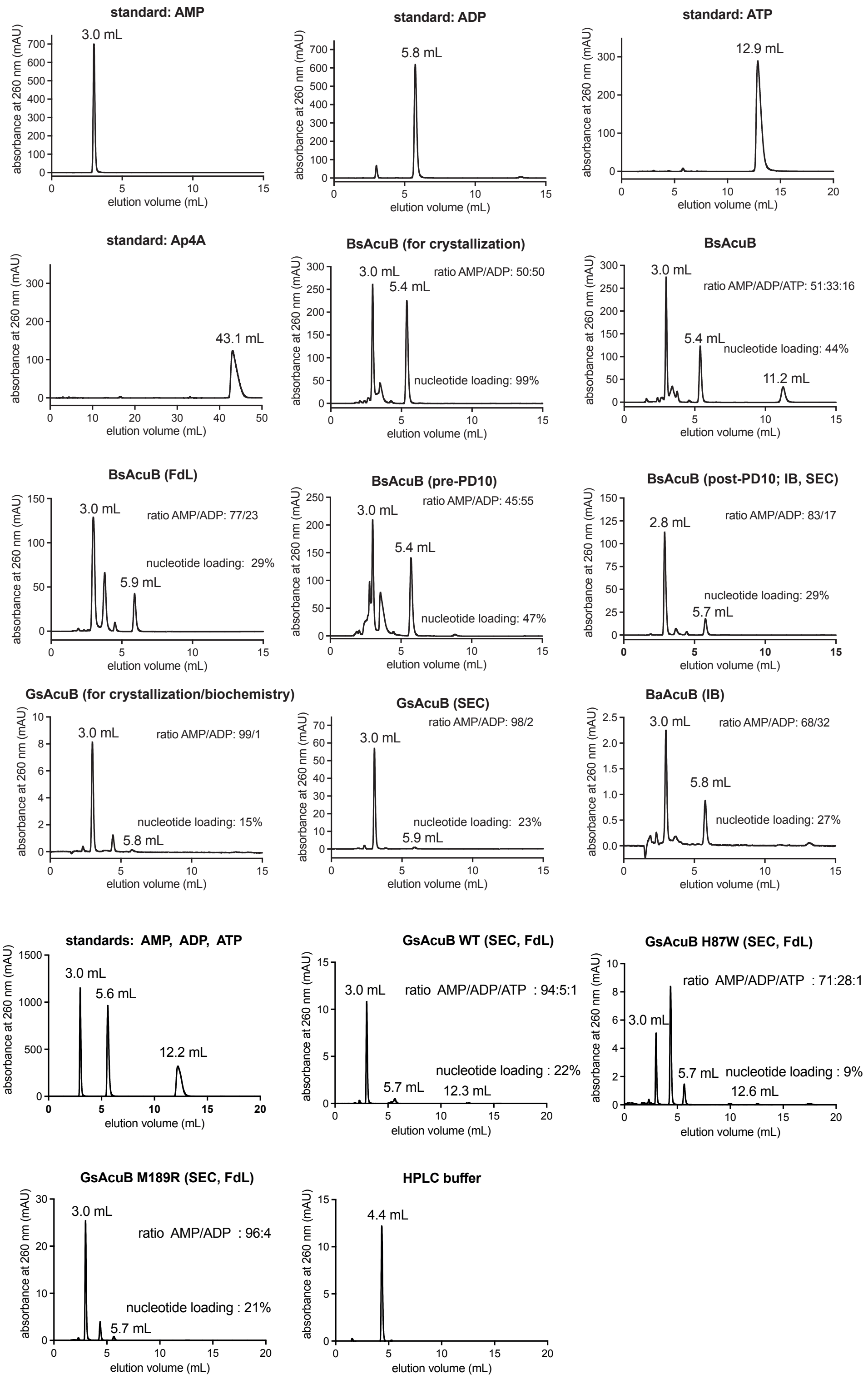


**Supplementary Fig. 3: HPLC-analyses of the nucleotide loading state of AcuB proteins analysed in this study.**

To separate different adenine-nucleotides a reversed-phase-C18 alkyl column was applied using a mobile phase containing acetonitrile and tetra-butyl-ammonium bromide (TBAB) as solvent. This allows to separate nucleotides with varying numbers of phosphoryl groups. The elution of the adenine-nucleotides was assessed by following the absorption at 260 nm ($A_{260\ nm}$). The proteins BsAcuB, BaAcuB and GsAcuB and different preparations thereof were analysed by HPLC. Standards using solutions of AMP, ADP, ATP and Ap4A were analysed to reveal the elution volume. Source data are provided as Source Data file.

**AcuB proteins:**
>sp|P39066|ACUB_BACSU Acetoin utilization protein AcuB OS=Bacillus subtilis (strain 168) OX=224308 GN=acuB PE=1 SV=1
MIVEQIMKRDVITLTKTDTLETAICKLKEFHIRHLPVVDEERHVIGMITDRDMKQASPSIFEENKRSLFLTRSVD
SIMKKDVVCAHPLDFVEEISAVFYEHGIGCLPVVHHQKLIGILTKTDLLRTFVKLTGADQPGSQIEIKVNDITKS
LAEISSLCQDLQVKILSVLVYPHDDPGVKVLVFRVKTMNPLPFLQALQRNGHHVVWPSEQRDLL

>tr|A0AAC8NB33|A0AAC8NB33_BACAN Acetoin utilization protein AcuB OS=Bacillus anthracis OX=1392 GN=acuB PE=4 SV=1
MIVEEIMNQDVVTLRPNDTIETAIRTIRTKGIRHIPIVDQNNHVVGIISDRDVRDASPSILDEQVSLDMLKQPLE
LIMKHPVMTCHPLDFVEEIATLFFENKIGCLPVTKAGKLVGIISESTVLHTLVKLTGAHQPSSQIEIQVKNEPGI
LGKVVAIFSELQINIVSVLVYPAKDENDKVLVFRIQTMNPLKVIDALEAEGYRVLWPNIMGMQA

>tr|A0AB37GSF4|A0AB37GSF4_BACLI Acetoin utilization AcuB family protein OS=Bacillus licheniformis OX=1402 GN=I6G80_09820 PE=4 SV=1
MIVEKIMKRDVVTLSRTDTIEEAIKRMRTFHIKHLPVINERGTVIGIVTDRDVKTASPSIFAQNRSNEDLKKPVE
LIMSKEVITGHPLDFAEEISAVFFEHEIGCLPIVKNGKLVGIVTKSDLLHTLVQLTGAHFPGSQIEIKTDHIARS
LADISAIFSRRQIAILSVLVYPGDEDDSKILVFRVQTMNPASIIKDVKSKGYQVLWPAVQRDLP

>UPI001F4752A3 | WP_237420712.1 Geobacillus stearothermophilus AcuB
MLVEQAMKAPVITLRATNTIAEALQLLRHHRIRHLPVVDGEGRLLGLVTSQDLRDASPSIFHLHEHLEDLQKPVS
TIMKTDLIVGHPLDFVEEVAALFYEHRIGCLPIVNHGKLVGIITQTDLLRTFIELTGVHQPGSQIEIKVPNEAGM
LSKAAAIISERHVNIASVLVYPAPDPNEKILVFRVQTMNPLPLIRDLQNAGYHVLWPNLPGVTS

**AcuC proteins:**
>sp|P39067|ACUC_BACSU Acetoin utilization protein AcuC OS=Bacillus subtilis (strain 168) OX=224308 GN=acuC PE=3 SV=1
MRDSVFIYSPSYQTYMFHQEHPFNQQRVLLTYDLLKTINAFDDGDIVTPRLASEEELSLVHTDDYIQAVKLAGAG
KLPAEEGESYGLGTEDTPVFAGMHEAASLLVGGTLTAADWVMSGQALHAANLGGGLHHGFRGRASGFCIYNDSAV
AIQYIQKKYSARVLYIDTDAHHGDGVQFTFYDNPDVCTLSIHETGRYLFPGTGQIQEKGSGKGYGYSFNIPLDAF
TEDDSFLEAYRTAASEVAAYFEPDVIISQNGADAHYYDPLTHLSATINIYEEIPRLAHTLAHQYCGGKWIAVGGG
GYDIWRVVPRAWARIWLEMKGIDPGHEIPPEWIVKWQKQCPVALPSSWSDPADLYPPIPRKPEITEKNAQTVSKA
LYAIRSEQQRTK

>tr|A0A6L7GXN4|A0A6L7GXN4_BACAN Acetoin utilization protein AcuC OS=Bacillus anthracis OX=1392 GN=acuC PE=3 SV=1
MSSAFIYSDDFRGYSFSPDHPFNQLRVTLTYDLLQKGGFISPSQIISPRMATDEEIAYIHTEEYINAVKRAGEGK
LEKSIAMTYGLGTEDTPMFPNMHEASALLVGGTLTAVDAVLSGKVKHALNLGGGLHHGFRGKASGFCIYNDSSIA
MKYIQKKYGLRVLYIDTDAHHGDGVQWSFYDDPNVCTISLHETGRYLFPGTGAVNERGQGNGYSYSFNVPLDAFT
EDESFLDSYRTVVKEVAAYFKPDIILTQNGADAHYYDPLTHLCATMNIYREIPKLAREIANEYCEGRWIAVGGGG
YDHWRVVPRAWALIWLEMNNIQNISGYLPPEWIDAWKGQAETELPLTWEDPNNMYKPIPRKPEIEEKNALTVAKS
LEIIRNNMKKSLY

r|Q65G31|Q65G31_BACLD Acetoin utilization protein AcuC OS=Bacillus licheniformis (strain ATCC 14580 / DSM 13 / JCM 2505 / CCUG 7422 / NBRC 12200 / NCIMB 9375 / NCTC 10341 / NRRL NRS-1264 / Gibson 46) OX=279010 GN=acuC PE=3 SV=1
MKDSVFIFSPSYQTYQFHQDHPFNQLRVYVTYDLLNTVGAFEPGETIAPRAATETELELVHTGDYIRAVQLAGAG
KLPAAESENYGLGTEDTPVFAGMHEAASLLVGGTLTAADYVMTGKARHALNLGGGLHHGFRGRASGFCVYNDSSV
VIRYLQKKYHAKVLYIDTDAHHGDGVQFTFYDDPSVCTVSIHETGRYLFPGTGQVQERGHGEGYGYAFNIPLDAF
TEDESFLDSYRTAVSEIAEFFKPDVILSQNGADAHYYDPLTHLCTTMKIYEEIPKLAHELAHTYCSGRWIAVGGG
GYDIWRVVPRAWARIWLEMKGMEPEEKMPEKWLKKWGKQASVRLPANWSDPEDLYPPIPRKTEITEKNAQTVAKA
LYPIRSAQRTT

>UPI001F1F0585 | WP_237420713.1 Geobacillus stearothermophilus AcuC
MSRNCAFVYSEQFLQYKFHDDHPFNQLRVKMTYDLLCTLGALEDRQIVAPRMATDDELALIHDRSYIEAVKAAGR
GELSEAAAQNYGLGTEDTPIFPNMHEASALLVGSTLTAVDAVLSGAAEHALNLGGGLHHGFRGKASGFCVYNDSA
VAIQYIREKYGLRVLYVDTDAHHGDGVQWAFYDDPNVCTFSIHETGRYLFPGTGNVNERGLGAGYGYSFNIPVDA
FTEDESWIAAYTTALREIADFFRPDVIVTQNGVDAHYYDPLTHLSVTMKTYRAIPKLAHEIAHEYCDGRWIAVGG
GGYDIWRVVPRAWALLWLEMTGQADVSGPLPDEWRERWQPLSPVALPLEWDDPDDLYPPIPRKAEISEKNAQTVE
KALYFIRSQRPAR

**AMP-forming acetyl-CoA synthetase from *B. subtilis*:**

```
>sp|P39062|ACSA_BACSU Acetyl-coenzyme A synthetase OS=Bacillus subtilis
(strain 168) OX=224308 GN=acsA PE=1 SV=1
MNLKALPAIEGDHNLKNYEETYRHFDWAEAEKHFSWHETGKLNAAYEAIDRHAESFRKNKVALYYKDAKRDEKYT
FKEMKEESNRAGNVLRRYGNVEKGDRVFIFMPRSPELYFIMLGAIKIGAIAGPLFEAFMEGAVKDRLENSEAKVV
VTTPELLERIPVDKLPHLQHVFVVGGEAESGTNIINYDEAAKQESTRLDIEWMDKKDGFLLHYTSGSTGTPKGVL
HVHEAMIQQYQTGKWVLDLKEEDIYWCTADPGWVTGTVYGIFAPWLNGATNVIVGGRFSPESWYGTIEQLGVNVW
YSAPTAFRMLMGAGDEMAAKYDLTSLRHVLSVGEPLNPEVIRWGHKVFNKRIHDTWWMTETGSQLICNYPCMDIK
PGSMGKPIPGVEAAIVDNQGNELPPYRMGNLAIKKGWPSMMHTIWNNPEKYESYFMPGGWYVSGDSAYMDEEGYF
WFQGRVDDVIMTSGERVGPFEVESKLVEHPAIAEAGVIGKPDPVRGEIIKAFIALREGFEPSDKLKEEIRLFVKQ
GLAAHAAPREIEFKDKLPKTRSGKIMRRVLKAWELNLPAGDLSTMED
```

**Lysine acetyltransferase AcuA from *B. subtilis*:**

```
>sp|P39065|ACUA_BACSU Acetoin utilization protein AcuA OS=Bacillus subtilis
(strain 168) OX=224308 GN=acuA PE=1 SV=1
MEHHKTYHSANIKTATGSLLIEGPVSPEDLAGYEFHKDLTAFRPPREQHEALVDIAGLPEGRIIIARDGRTIVGY
VTYLYPDPLERWSEGNMEDLIELGAIEVAPDYRGCAVGKTLLTVSMMDEQMENYIVMTTEYYWHWDLKGMKKDVW
EYRKIMEKMMNAGGLVWFATDEPEISSHPANCLMARIGKNVSQESIEQFDRLRFYHRYMY
```

**GTP-binding protein Ran from *Homo sapiens*:**

```
>sp|P62826|RAN_HUMAN GTP-binding nuclear protein Ran OS=Homo sapiens
OX=9606 GN=RAN PE=1 SV=3
MAAQGEPQVQFKLVLVGDGGTGKTTFVKRHLTGEFEKKYVATLGVEVHPLVFHTNRGPIKFNVWDTAGQEKFGGL
RDGYYIQAQCAIIMFDVTSRVTYKNVPNWHRDLVRVCENIPIVLCGNKVDIKDRKVKAKSIVFHRKKNLQYYDIS
AKSNYNFEKPFLWLARKLIGDPNLEFVAMPALAPPEVVMDPALAAQYEHDLEVAQTTALPDEDDDL
```

**Transcriptional regulator RutR from *Escherichia coli*:**

```
>sp|P0ACU2|RUTR_ECOLI HTH-type transcriptional regulator RutR
OS=Escherichia coli (strain K12) OX=83333 GN=rutR PE=1 SV=1
MTQGAVKTTGKRSRAVSAKKKAILSAALDTFSQFGFHGTRLEQIAELAGVSKTNLLYYFPSKEALYIAVLRQILD
IWLAPLKAFREDFAPLAAIKEYIRLKLEVSRDYPQASRLFCMEMLAGAPLLMDELTGDLKALIDEKSALIAGWVK
SGKLAPIDPQHLIFMIWASTQHYADFAPQVEAVTGATLRDEVFFNQTVENVQRIIIEGIRPR
```

**Supplementary Figure 4: Sequences for the proteins used in this study.**
All protein sequences used for alignments and all experiments in this study were obtained from UniProt database with the following accession codes: *Bacillus subtilis* AcuB P39066; *Bacillus anthracis* AcuB A0AAC8NB33; *Bacillus licheniformis* AcuB A0AB37GSF4; *Geobacillus stearothermophilus* AcuB WP_237420712.1; *Bacillus subtilis* AcuC P39067; *Bacillus anthracis* AcuC A0A6L7GXN4; *Bacillus licheniformis* AcuC Q65G31; *Geobacillus stearothermophilus* AcuC WP_237420713.1. *Bacillus subtillis* AcsA P39062; *Bacillus subtilis* AcuA P39065; *Homo sapiens* Ran P62826; *Escherichia coli* RutR P0ACU2.

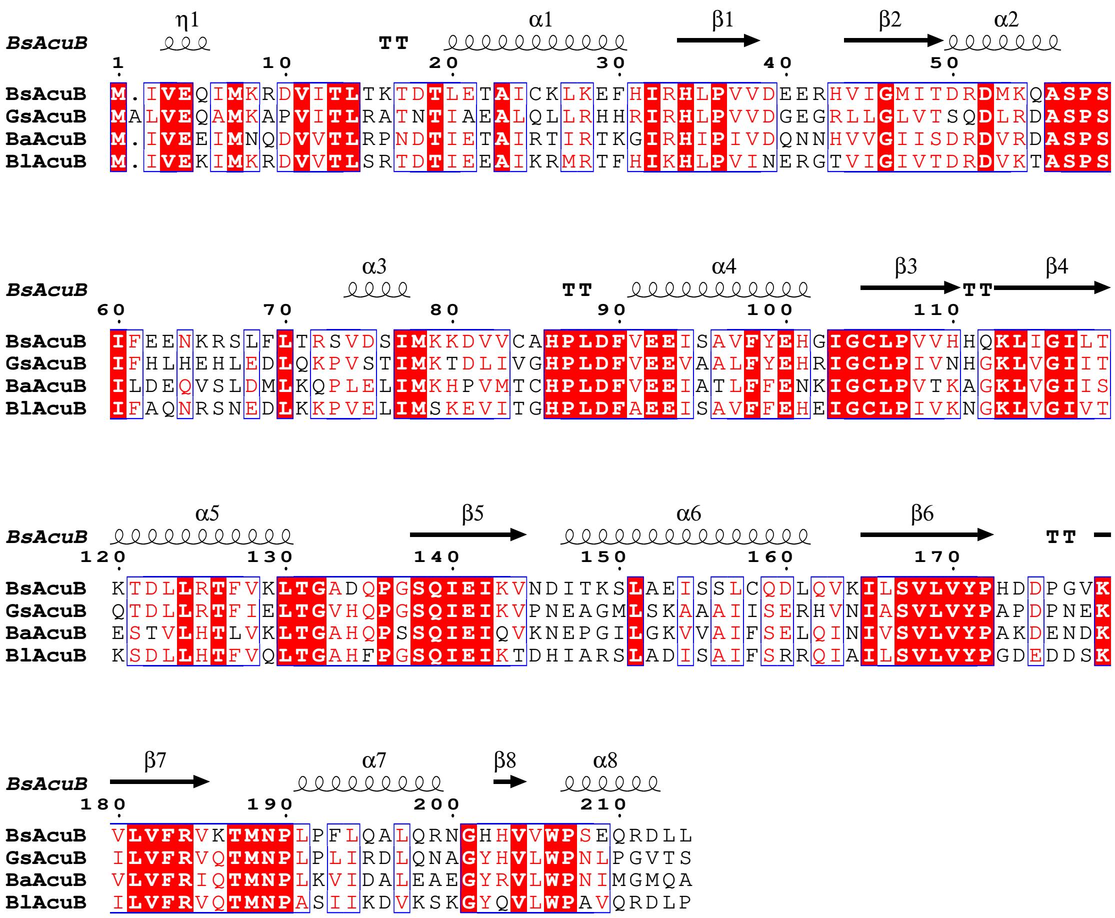

**Supplementary Fig. 5: Primary sequence alignment for AcuB proteins from different Bacilli.** All protein sequences used for alignments were obtained from UniProt database with the following accession codes: *Bacillus subtilis* AcuB P39066; *Bacillus anthracis* AcuB A0AAC8NB33; *Bacillus licheniformis* AcuB A0AB37GSF4; *Geobacillus stearothermophilus* AcuB WP_237420712.1. Sequence alignments were conducted with the multiple sequence alignment (MSA) program Clustal Omega (v0(1.2.4)). Further analysis was done using the software ESPript3.0 (v3.0.10)[2,3]. The crystal structure of *B. subtilis* AcuB, i.e. BsAcuB (PDB: 9SAV), was used for plotting the secondary structure elements above the alignment.

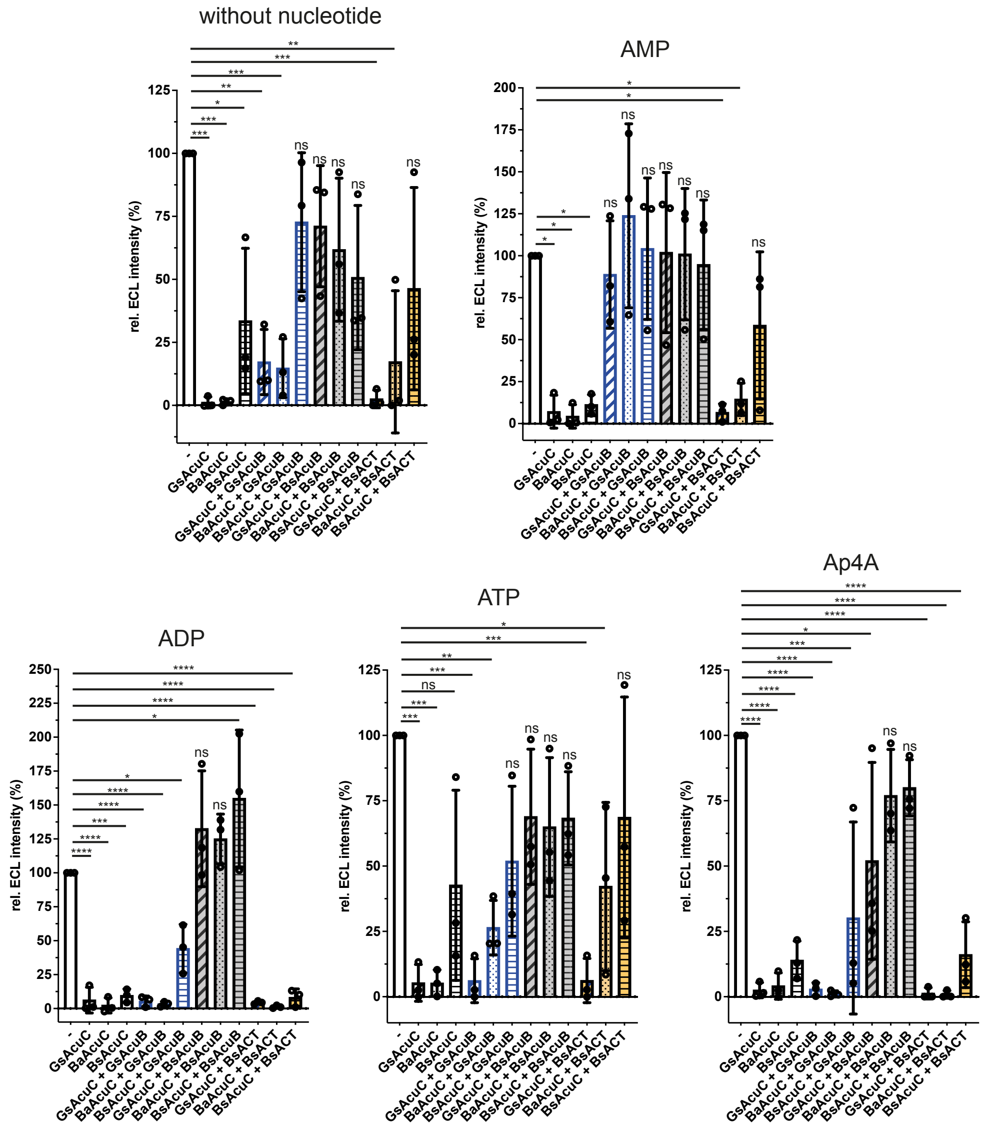


**Supplementary Fig. 6: Quantification of the Immunoblots shown in Fig. 2.** The signal obtained for the anti-acetyl-lysine (IB: anti-AcK) blot was normalized by the signal obtained for the loading control (LC: PoS). The experiment was performed in three independent replicates (*n*=3), one example is shown. Data are presented as mean ± SD. The statistical analyses were performed conducting an ordinary ANOVA test ($p<0.05$: *; $p<0.01$: **; $p<0.001$: ***; $p<0.0001$:****). Source data are provided as Source Data file.

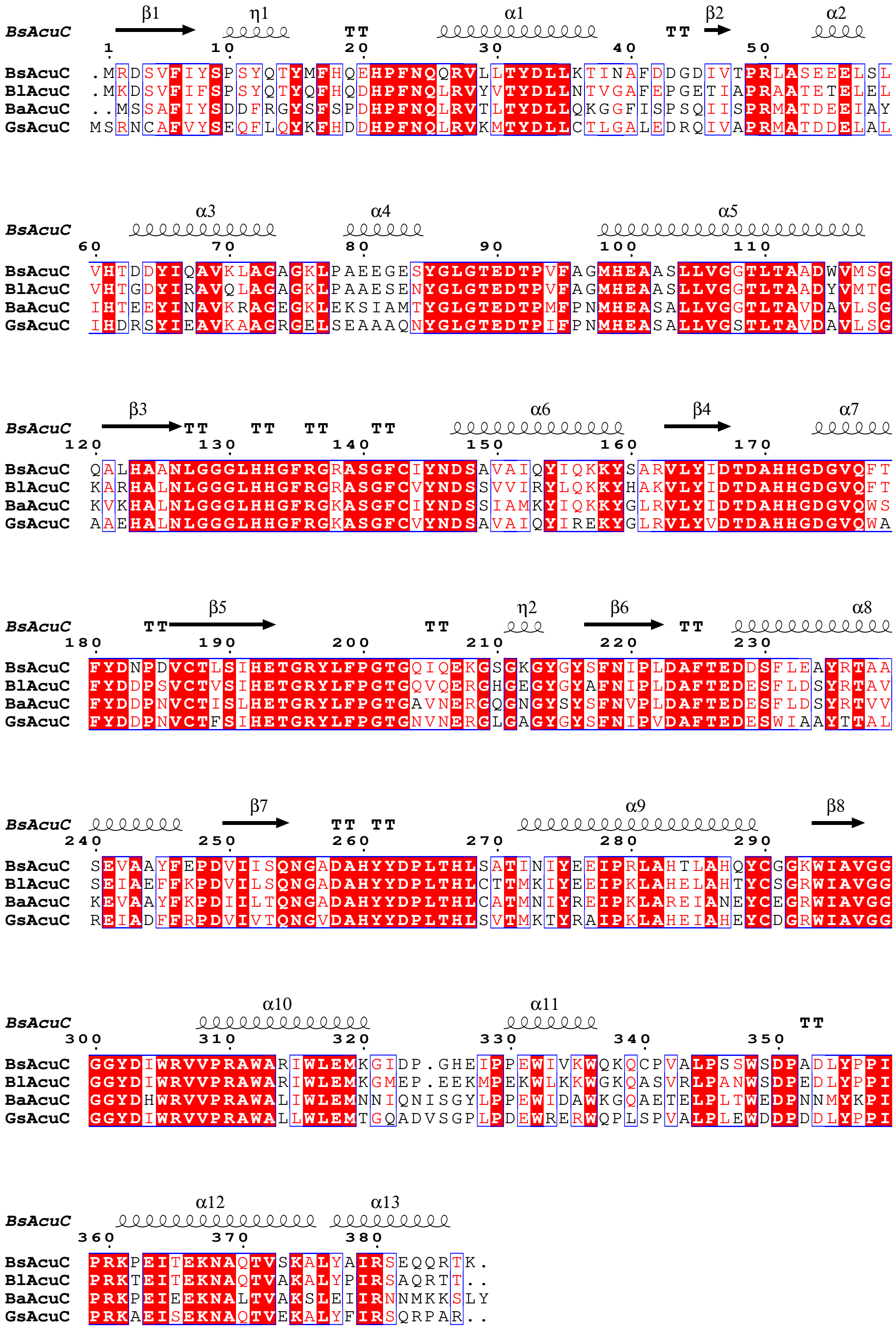

**Supplementary Fig. 7: Primary sequence alignment for AcuC proteins from different Bacilli analyzed in this study.**

All protein sequences used for alignments were obtained from UniProt database with the following accession codes: *Bacillus subtilis* AcuC P39067; *Bacillus anthracis* AcuC A0A6L7GXN4 ; *Bacillus licheniformis* AcuC Q65G31; *Geobacillus stearothermophilus* AcuC WP_237420713.1. Sequence alignments were conducted with the multiple sequence alignment (MSA) program Clustal Omega (v0(1.2.4)). Further analysis was done using the software ESPript3.0 (v3.0.10)[2,3]. The AlphaFold3 model of the structure of *B. subtilis* AcuC, i.e. BsAcuC, was used for plotting the secondary structure elements above the alignment.

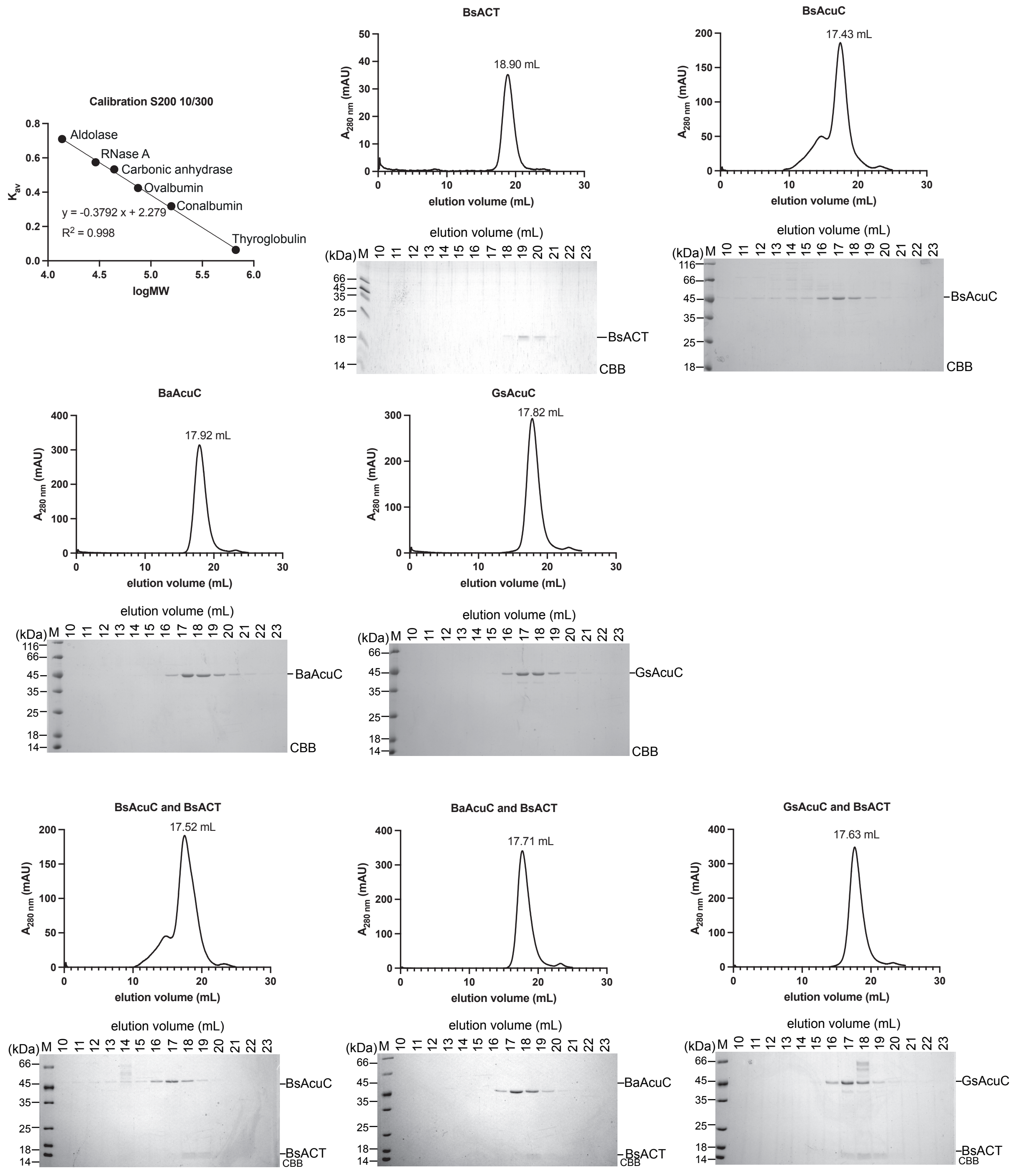


**Supplementary Fig. 8: Analysis of complex formation of BsACT domain and BsAcuC, BaAcuC, and GsAcuC by analytical SEC on an S200 10/300 column.** For all runs no complex formation between the analysed AcuC proteins and BsACT is detectable indicating a moderate affinity in the micromolar range of BsACT towards these AcuC proteins. Elution was followed by recording the absorption at 280 nm ($A_{280\ nm}$) in milli absorbance units (mAU). The fractions of the analytical SEC were analyzed by SDS-PAGE. The lane labelled with M represents the protein molecular weight marker. The SDS-PAGE gels were stained with Coomassie-brilliant blue (CBB). Source data are provided as Source Data file.

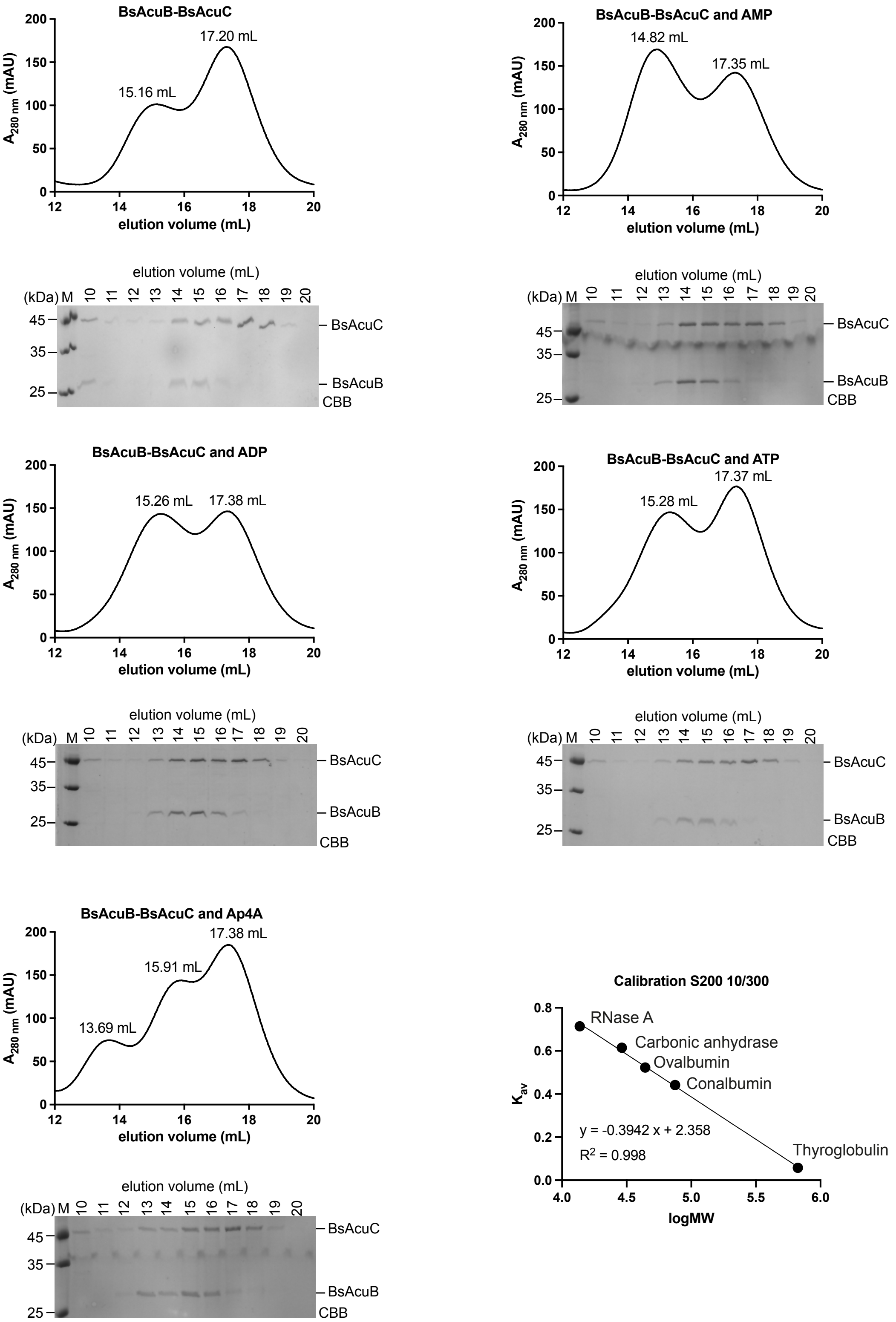


**Supplementary Fig. 9: Analysis of complex formation of BsAcuB and BsAcuC in dependence of presence of nucleotides AMP, ADP, ATP and Ap4A by analytical SEC on a S200 10/300 column.** For all runs complex formation between BsAcuC and BsAcuB is detectable. Even without addition of adenine nucleotides BsAcuC and BsAcuB form a complex. This is due to the application of 29% predominantly with AMP loaded BsAcuB protein. Upon addition of AMP a strong increase in BsAcuC•BsAcuB is visible. Elution was followed by recording the absorption at 280 nm ($A_{280\ nm}$) in milli absorbance units (mAU). The fractions of the analytical SEC were analyzed by SDS-PAGE. The lane labelled with M represents the protein molecular weight marker. The SDS-PAGE gels were stained with Coomassie-brilliant blue (CBB). Source data are provided as Source Data File Source data are provided as Source Data file.

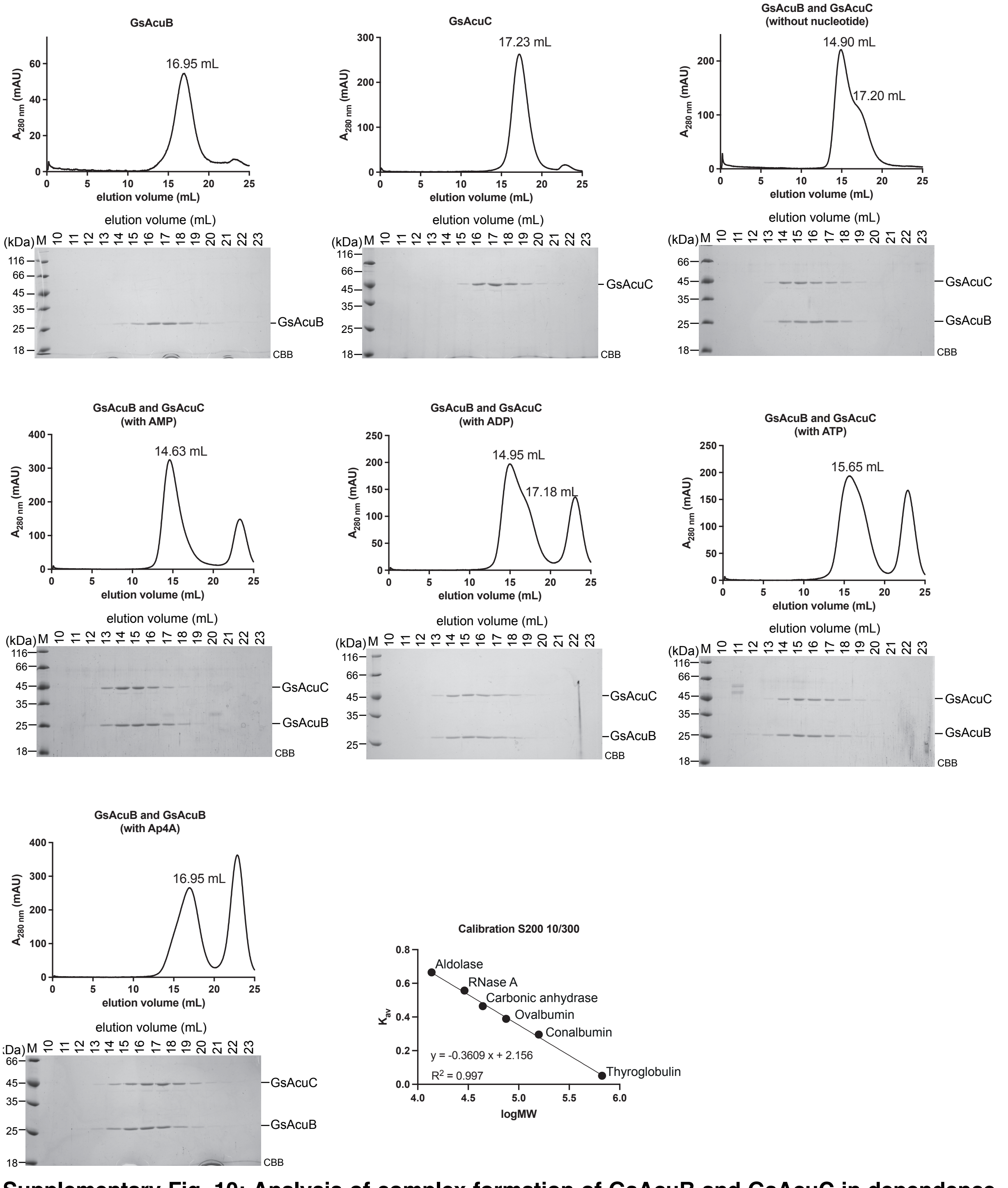


**Supplementary Fig. 10: Analysis of complex formation of GsAcuB and GsAcuC in dependence of presence of nucleotides AMP, ADP, ATP and Ap4A by analytical SEC on an S200 10/300 column.** For all runs complex formation between GsAcuC and GsAcuB is detectable. Even without addition of adenine nucleotides GsAcuC and GsAcuB form a complex. This is due to the application of only 15% predominantly with AMP loaded GsAcuB protein. Upon addition of AMP a strong increase in GsAcuC•GsAcuB is visible. Elution was followed by recording the absorption at 280 nm ($A_{280\ nm}$) in milli absorbance units (mAU). The fractions of the analytical SEC were analyzed by SDS-PAGE. The lane labelled with M represents the protein molecular weight marker. The SDS-PAGE gels were stained with Coomassie-brilliant blue (CBB). Source data are provided as Source Data file.

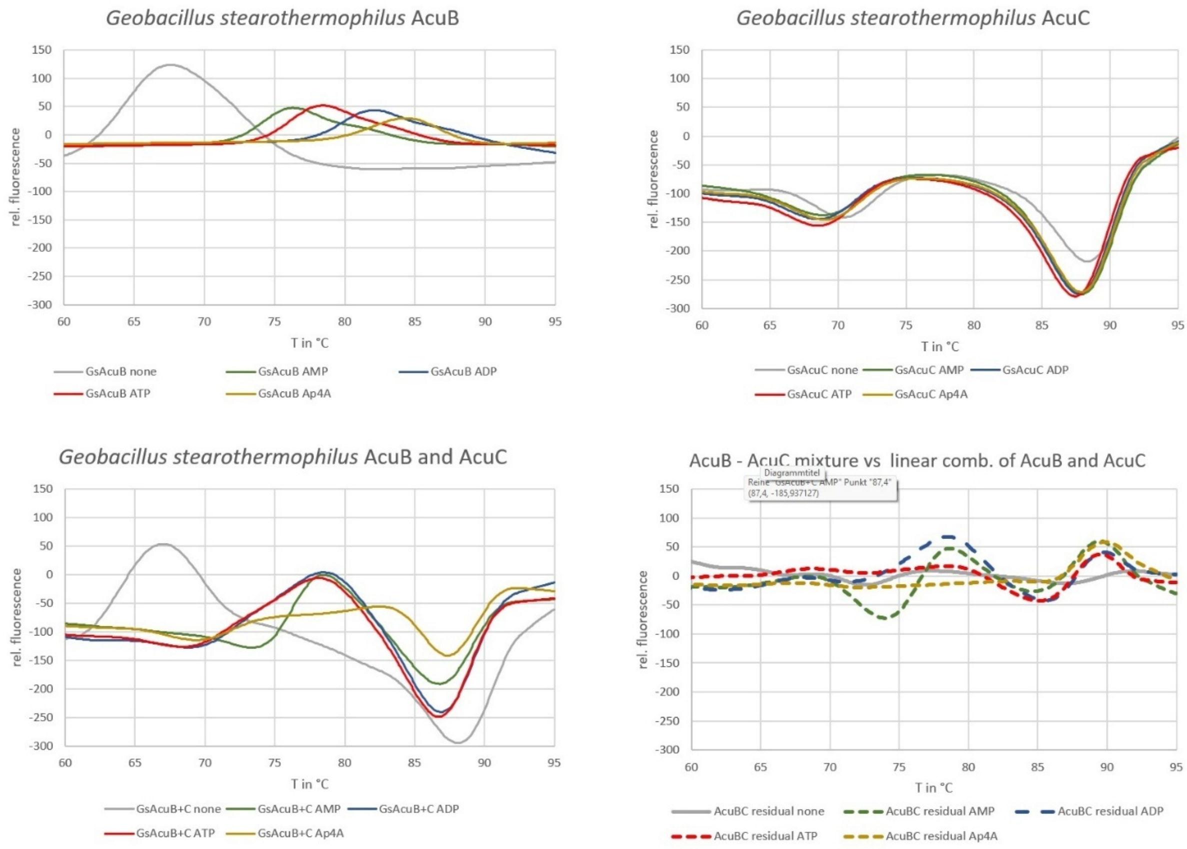


**Supplementary Fig. 11: Analysis of complex formation of GsAcuB and GsAcuC in dependence of presence of nucleotides AMP, ADP, ATP and Ap4A by nanoDSF.** AcuB, AcuC and an AcuB+AcuC mixture were measured by nanoDSF. The first derivatives of the fluorescence intensities at 330 nm are shown (average of 3 measurements). Data at 350 nm are very similar, but not shown. The mixture was fitted by a linear combination of the individual AcuB and AcuC data. The difference between the mixture and the fit is shown as grey line (without nucleotide) and dashed curves (with nucleotide). AcuB denaturation curves differ strongly depending on added nucleotides, whereas AcuC does not. Addition of all tested adenine nucleotides results in AcuB-AcuC interactions, whereas AcuB and AcuC do not interact in nucleotide free solution.

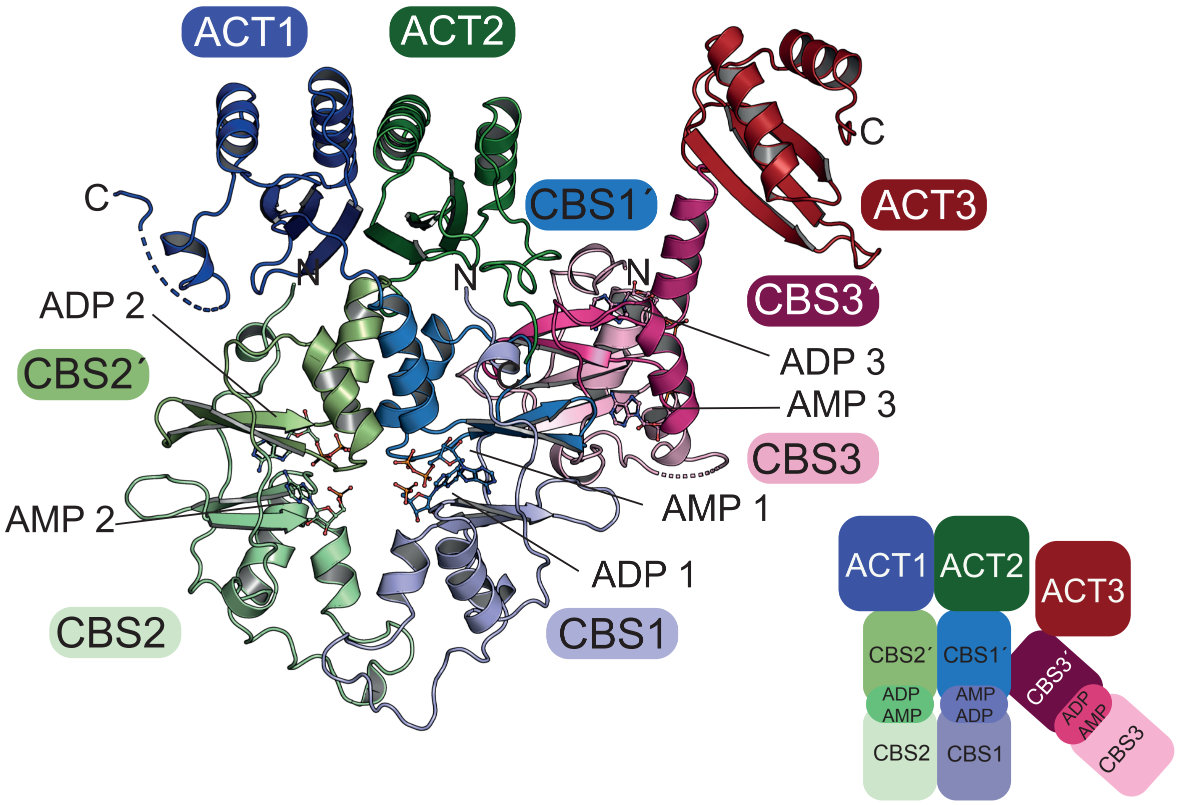


**Supplementary Fig. 12: Components of the asymmetric unit (AU) of the BsAcuB structure.** The crystals belong to the orthorhombic space group $P2_122_1$. Two monomers, chains A and B, form a dimer related by non-crystallographic symmetry (NCS). Inspection of these molecules reveal that they form an asymmetric dimer as both monomers show conformational differences. The third monomer (chain C) forms a crystallographic dimer directly lying on a crystallographic twofold symmetry axis. The figure was created with PyMOL[1].

a

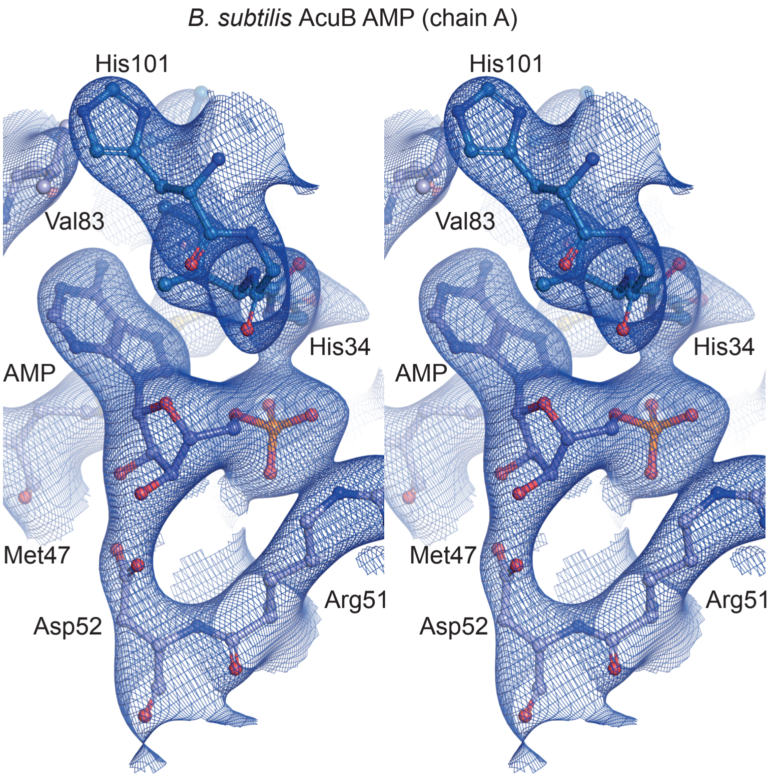


b

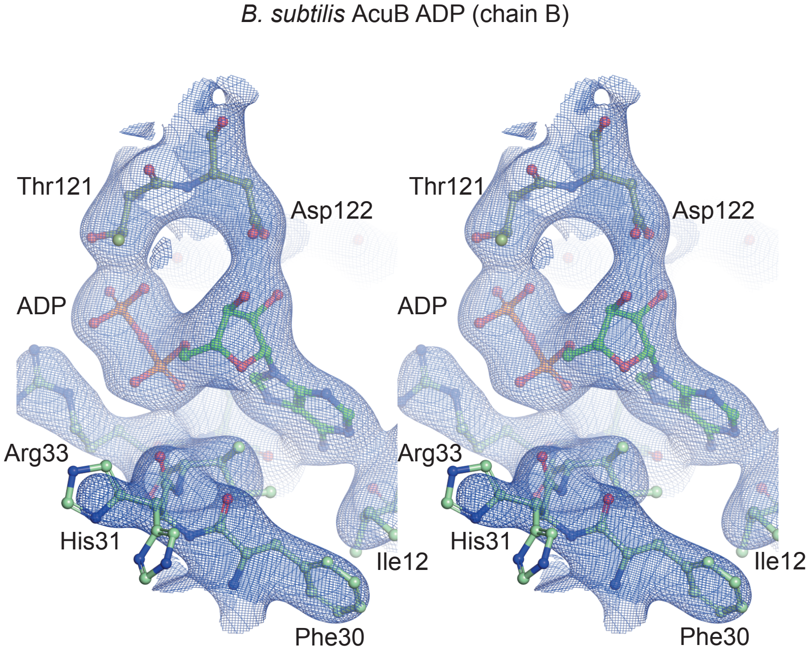


**Supplementary Fig. 13: Shown is a closeup of 2Fo-Fc electron density the type I and type II adenine nucleotide binding site of monomer A countered at 1σ obtained for the crystal structure of BsAcuB in complex with AMP and ADP (PDB: 9SAV [https://doi.org/10.2210/pdb9SAV/pdb]).** The figure was created with PyMOL[1].

Omit map of AMP and ADP nucleotides bound to *B. subtilis* AcuB

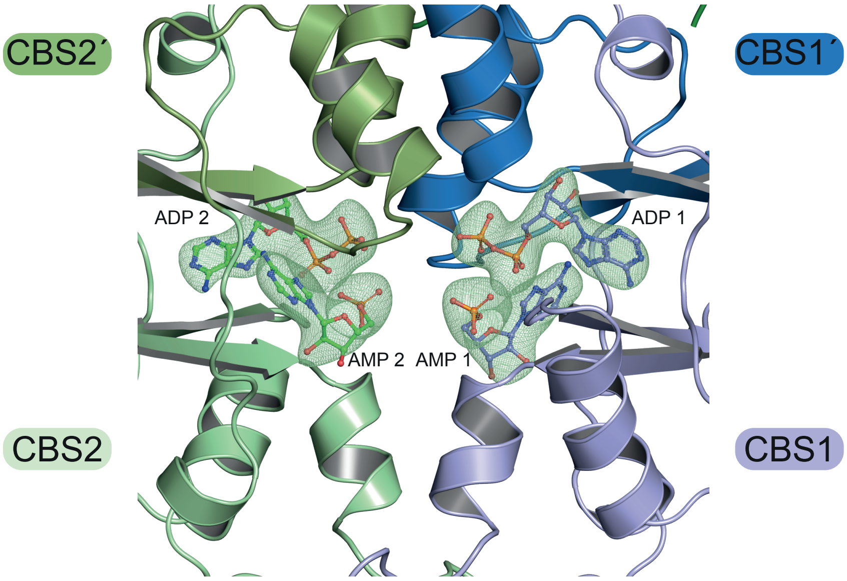


**Supplementary Fig. 14: Fo-Fc omit map countered at $3\sigma$ for adenine nucleotides bound to AcuB as indicated for the crystal structure of BsAcuB in complex with AMP and ADP (PDB: 9SAV [https://doi.org/10.2210/pdb9SAV/pdb]).** The figure was created with PyMOL[1].

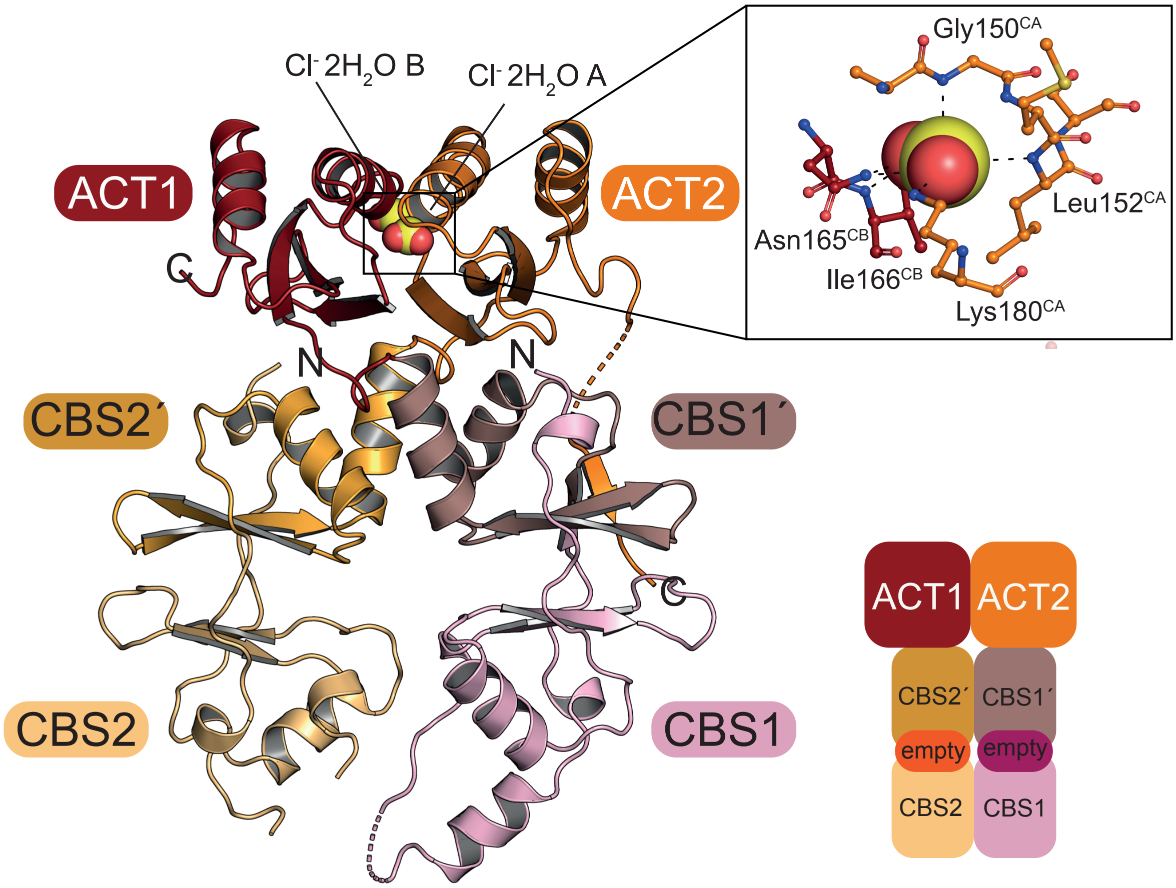


**Supplementary Fig. 15: Crystal structure of nucleotide free GsAcuB (PDB: 9SAW [https://doi.org/10.2210/pdb9SAW/pdb]).** Shown is a cartoon representation of the structure of nucleotide free GsAcuB. The figure was created with PyMOL[1].

a

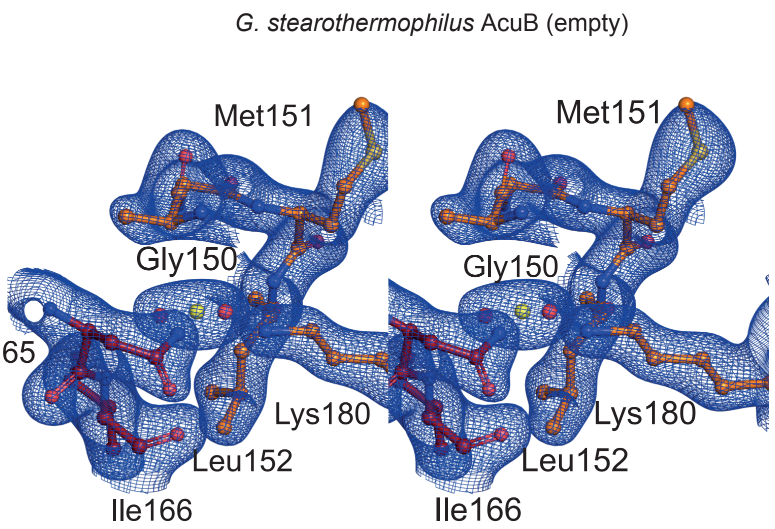


b

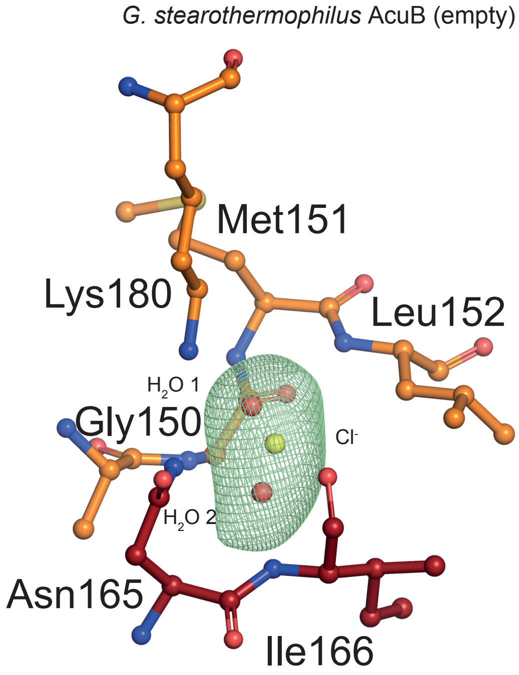


**Supplementary Fig. 16: Representative electron density obtained for the crystal structure of nucleotide free GsAcuB (PDB: 9SAW [https://doi.org/10.2210/pdb9SAW/pdb]).**

**a.** Shown is a closeup of 2Fo-Fc electron density the type I and type II adenine nucleotide binding site of monomer A countered at 1σ. The figure was created with PyMOL[1].

**b.** Fo-Fc omit map countered at 3σ for adenine nucleotides bound to AcuB as indicated. The figure was created with PyMOL[1].

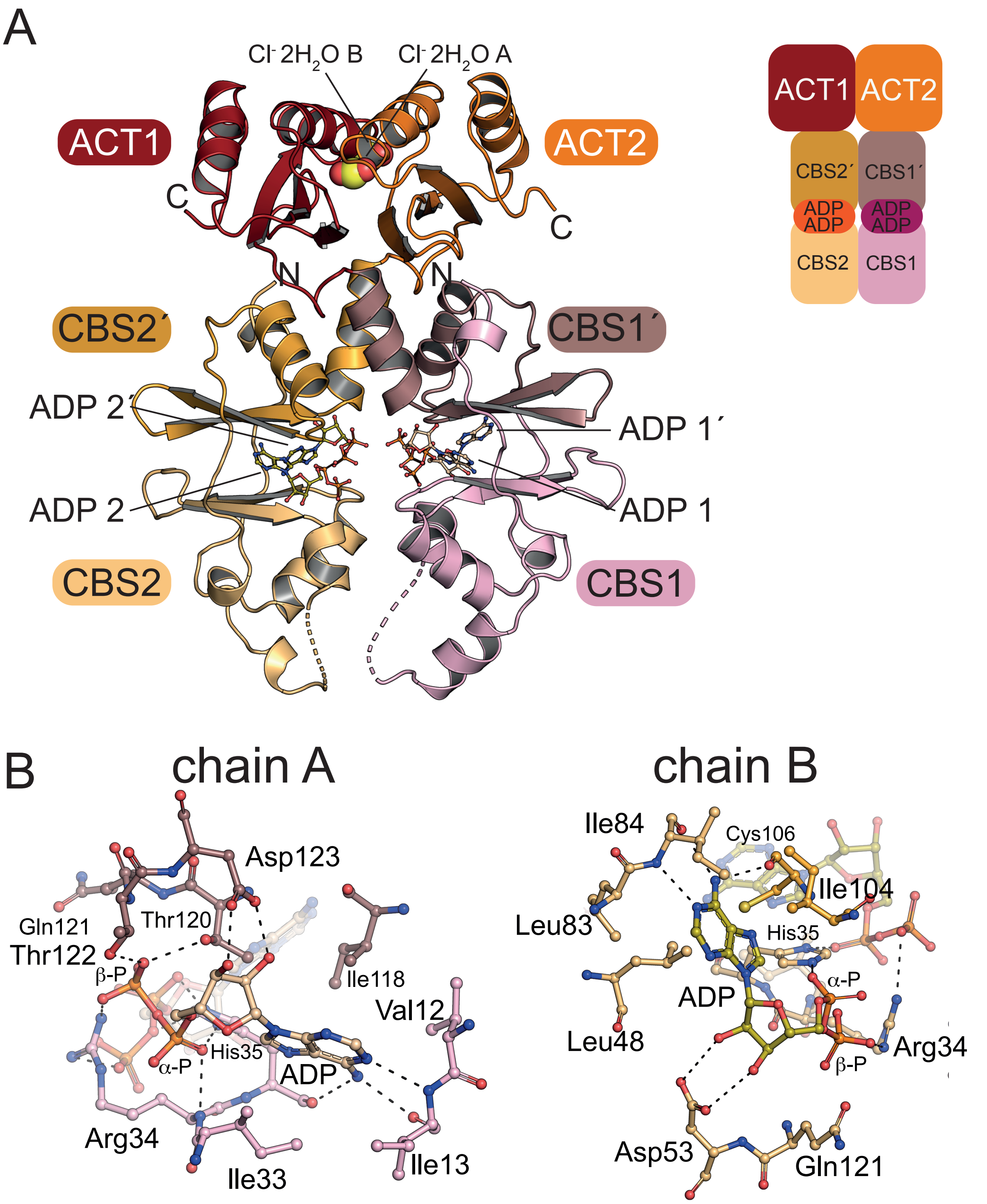


**Supplementary Fig. 17: Crystal structure of GsAcuB in complex with ADP (PDB: 9SAX [https://doi.org/10.2210/pdb9SAX/pdb]).**

**a.** Shown is a cartoon representation of the structure of GsAcuB in complex with four molecules of ADP bound to type I and type II binding sites. The figure was created with PyMOL[1].

**b.** Closeup of the type I adenine nucleotide binding site of monomer A and the type II binding site of monomer B. Both adenine nucleotide binding sites bind to ADP using the interactions as described in the manuscript text. The figure was created with PyMOL[1].

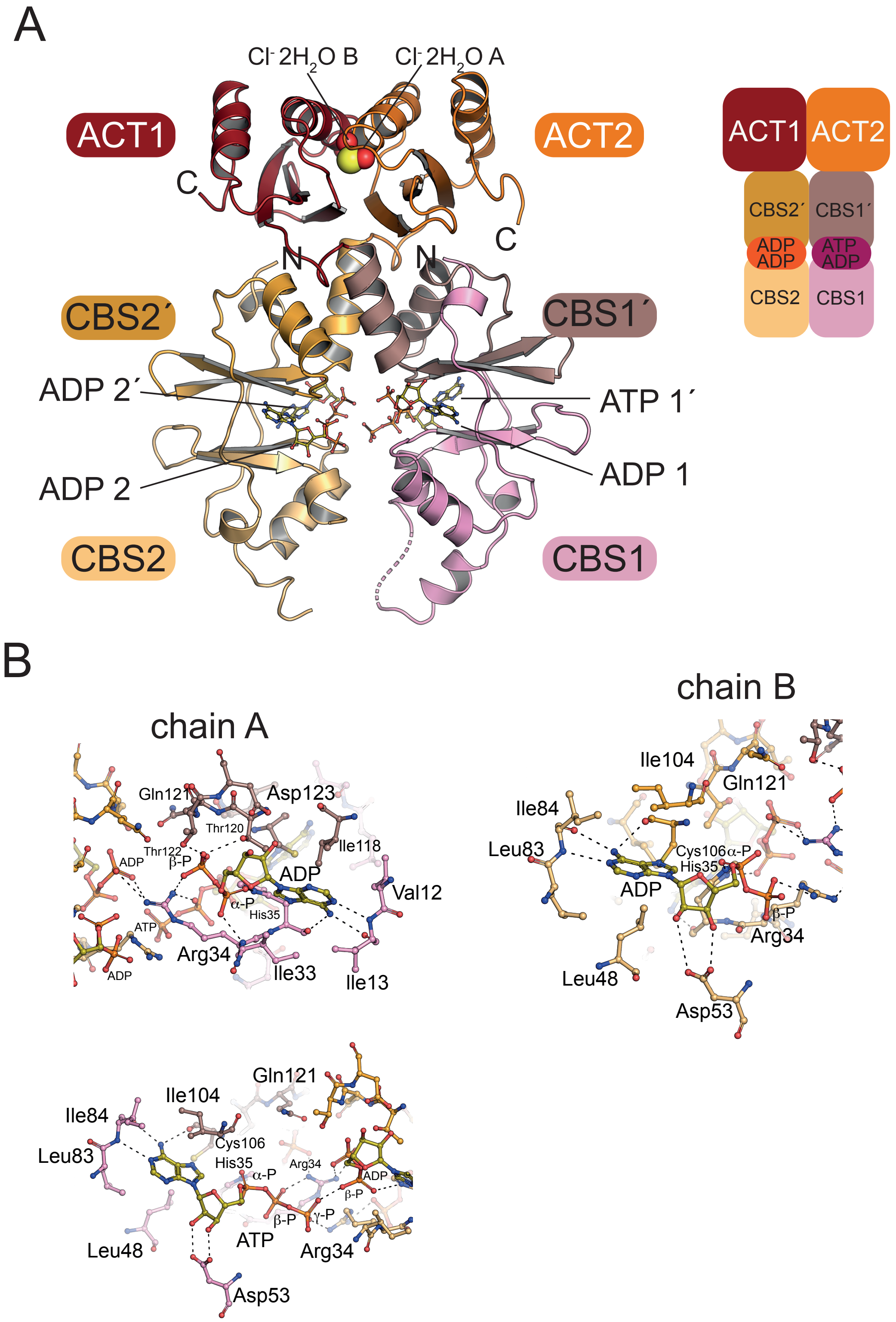


**Supplementary Fig. 18: Crystal structure of GsAcuB in complex with ADP and ATP (PDB: 9S51 [https://doi.org/10.2210/pdb9S51/pdb]).**

**a.** Shown is a cartoon representation of the structure of GsAcuB in complex with three molecules of ADP and one molecule of ATP. The ATP molecule is bound to the type II binding site in monomer A. The other type II binding site in monomer B and both of the type I binding sites are occupied by ADP molecules. The figure was created with PyMOL[1].

**b.** Closeup of the type I adenine nucleotide binding site of monomer A and the type II binding site of monomer B both occupied by ADP (upper panels). The ATP molecule occupies the type II adenine nucleotide binding site of monomer A (lower panel). The interactions of the bound adenine nucleotides with the AcuB protein are as described in the manuscript text. The figure was created with PyMOL[1].

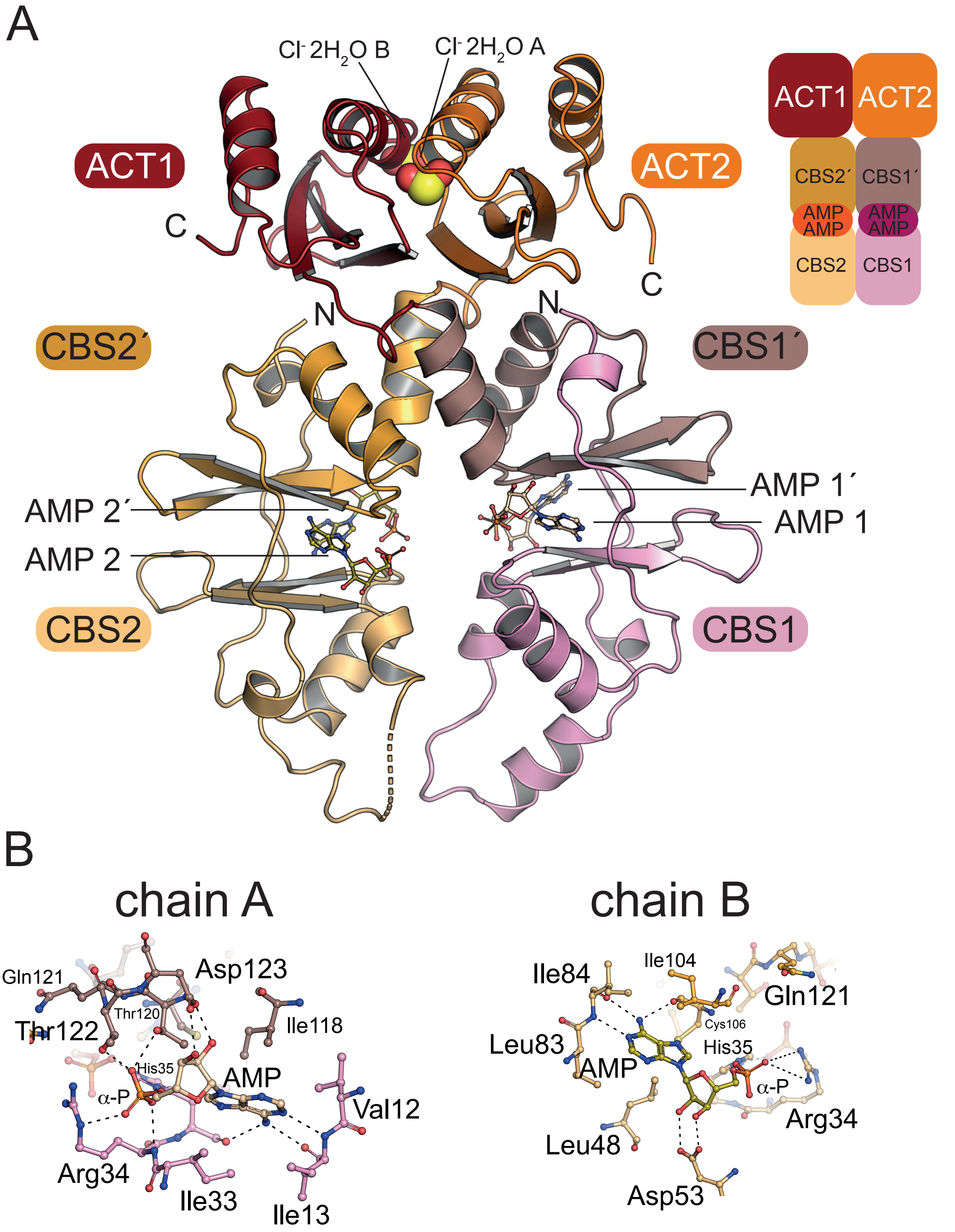


**Supplementary Fig. 19: Crystal structure of GsAcuB in complex with AMP (PDB: 9S52 [https://doi.org/10.2210/pdb9S52/pdb]).**

**a.** Shown is a cartoon representation of the structure of GsAcuB in complex with four molecules of AMP. The AMP molecules occupy all adenine nucleotide binding sites. The figure was created with PyMOL[1].

**b.** Closeup of the type I adenine nucleotide binding site of monomer A and the type II binding site of monomer B both occupied by AMP. The interactions of the bound AMP nucleotides with the AcuB protein are as described in the manuscript text. The figure was created with PyMOL[1].

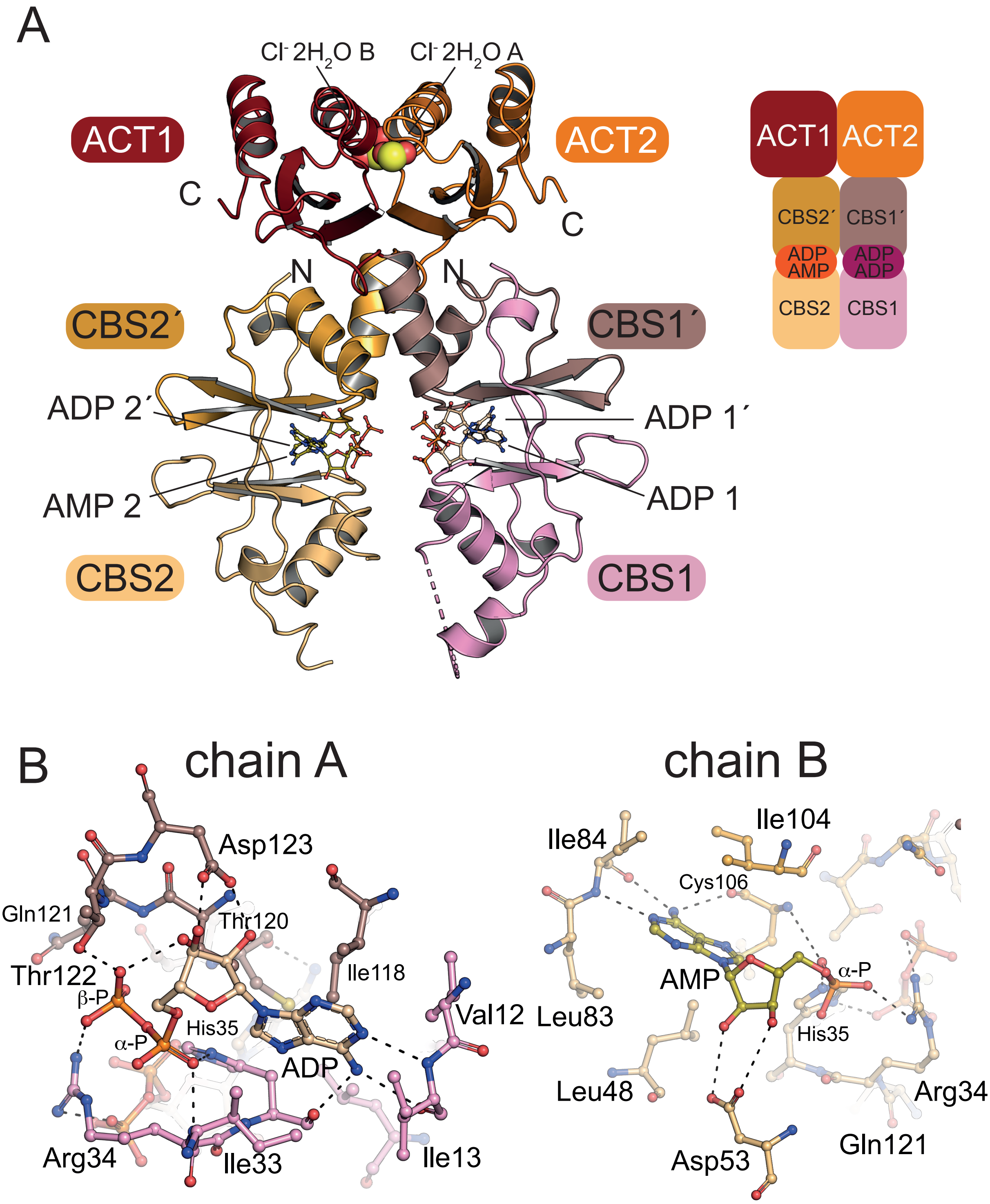


**Supplementary Fig. 20: Crystal structure of GsAcuB soaked with AMP and ATP (PDB: 9S4Z)** [https://doi.org/10.2210/pdb9S4Z/pdb]**.**

**a.** Shown is a cartoon representation of the structure of GsAcuB in complex with three molecules of ADP and one molecule of AMP. Although the crystals were soaked with AMP and ATP only three molecules of ADP and one molecule of AMP was visible in the crystals structure. The AMP molecule is bound to the type I binding site of monomer B. The other type I binding site in monomer A and both of type II binding sites are occupied by ADP molecules. The figure was created with PyMOL[1].

**b.** Closeup of the type I adenine nucleotide binding site of monomer A and the type II binding site of monomer B both occupied by ADP (upper panels). The ATP molecule occupies the type II adenine nucleotide binding site of monomer A (lower panel). The interactions of the bound adenine nucleotides with the AcuB protein are as described in the manuscript text. The figure was created with PyMOL[1].

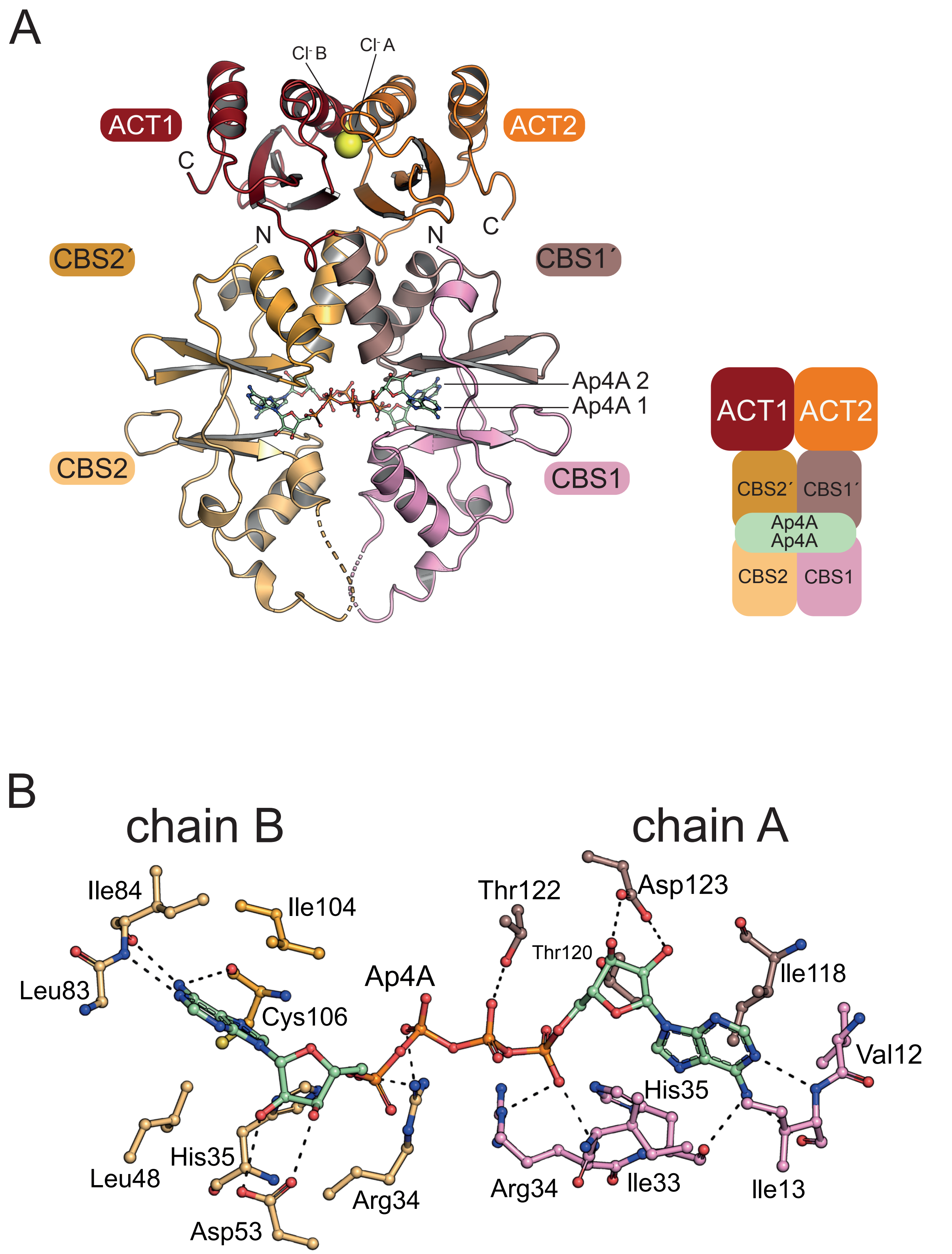


**Supplementary Fig. 21: Crystal structure of GsAcuB in complex with Ap4A (PDB: 9SAY [https://doi.org/10.2210/pdb9SAY/pdb]).**

**a.** Shown is a cartoon representation of the structure of GsAcuB in complex with two molecules of Ap4A. The two Ap4A molecules occupy all adenine nucleotide binding sites. Each Ap4A molecule binds at one site of the AcuB dimer thereby bridging both monomers. The figure was created with PyMOL[1].

**b.** Closeup of the type I adenine nucleotide binding site of monomer A and the type II binding site of monomer B both occupied by Ap4A. The interactions of the bound Ap4A nucleotides with the AcuB protein are as described in the manuscript text. The figure was created with PyMOL[1].

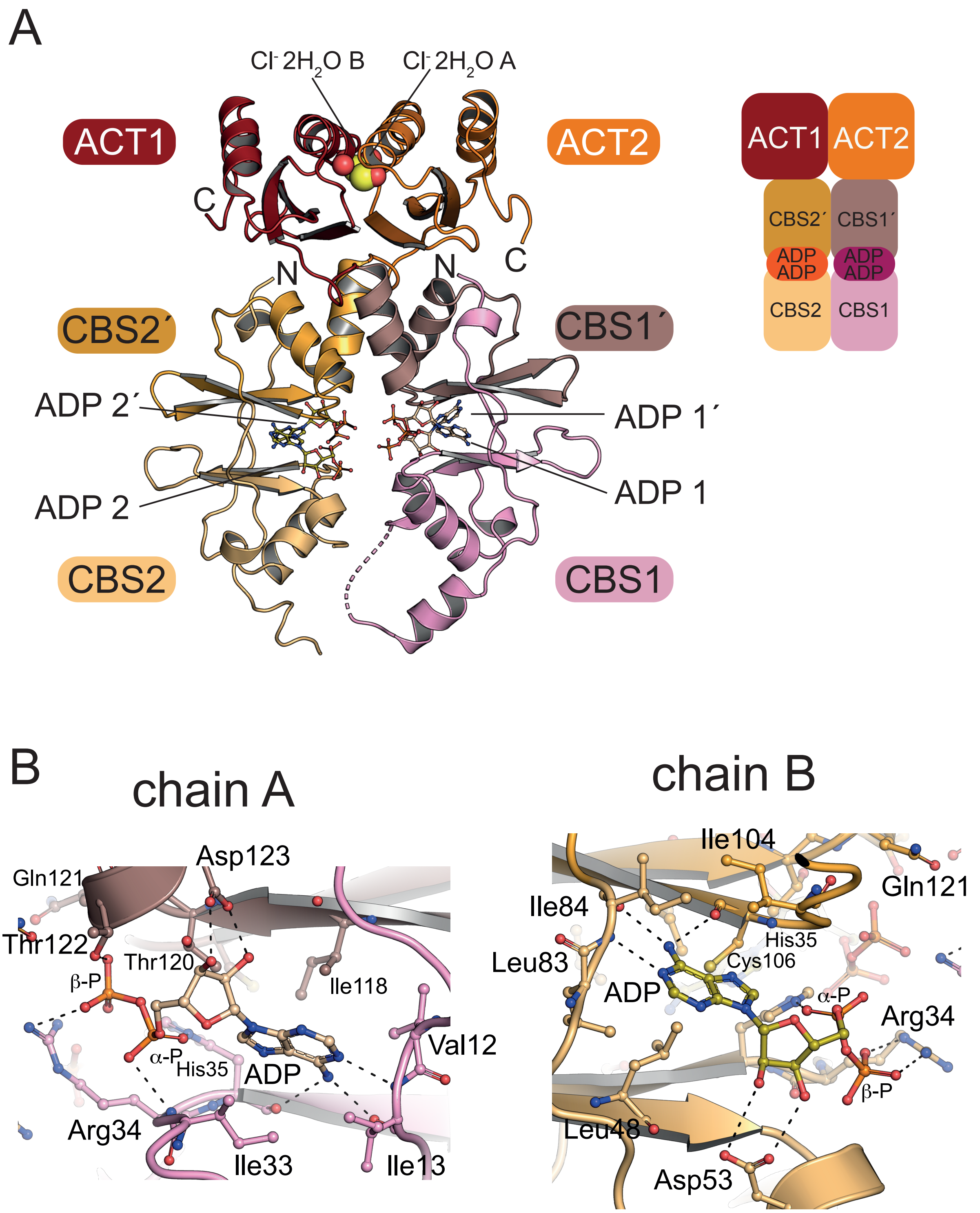


**Supplementary Fig. 22: Crystal structure of GsAcuB soaked with AMP and ATP (PDB: 9S50 [https://doi.org/10.2210/pdb9S50/pdb]).**

**a.** Shown is a cartoon representation of the structure of GsAcuB in complex with three molecules of ADP and one molecule of AMP. The AMP molecule is bound to the type I binding site of monomer B. The other type I binding site in monomer A and both of type I binding sites are occupied by ADP molecules. The figure was created with PyMOL[1].

**b.** Closeup of the type I adenine nucleotide binding site of monomer A and the type II binding site of monomer B both occupied by ADP (upper panels). The ATP molecule occupies the type II adenine nucleotide binding site of monomer A (lower panel). The interactions of the bound adenine nucleotides with the AcuB protein are as described in the manuscript text. The figure was created with PyMOL[1].

a

*G. stearothermophilus* AcuB (AMP)

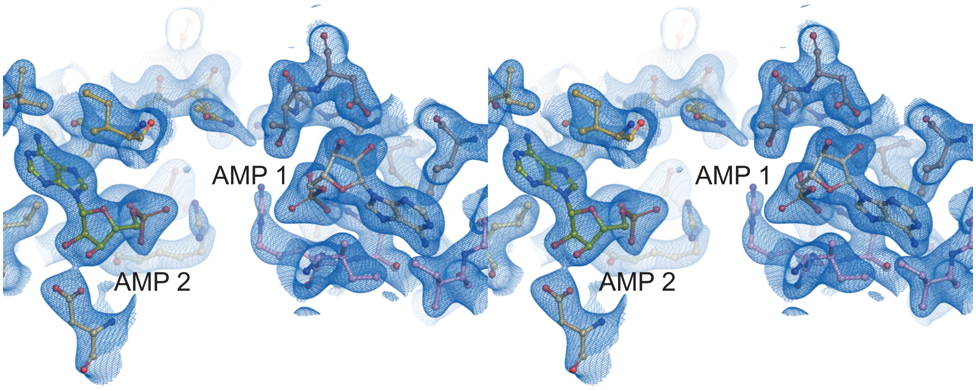


b

*G. stearothermophilus* AcuB (AMP)

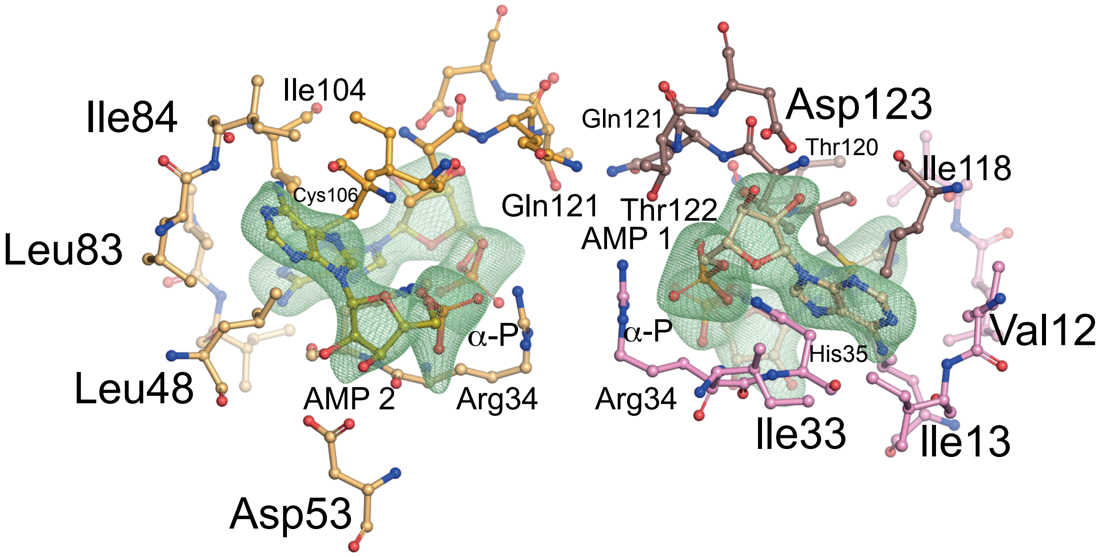


**Supplementary Fig. 23: Representative electron density obtained for the crystal structure of GsAcuB in complex with AMP (PDB: 9S52 [https://doi.org/10.2210/pdb9S52/pdb]).**

**a.** Shown is a closeup of 2Fo-Fc electron density the type I and type II adenine nucleotide binding site of monomer A countered at 1σ. The figure was created with PyMOL[1].

**b.** Fo-Fc omit map countered at 3σ for adenine nucleotides bound to AcuB as indicated. The figure was created with PyMOL[1].

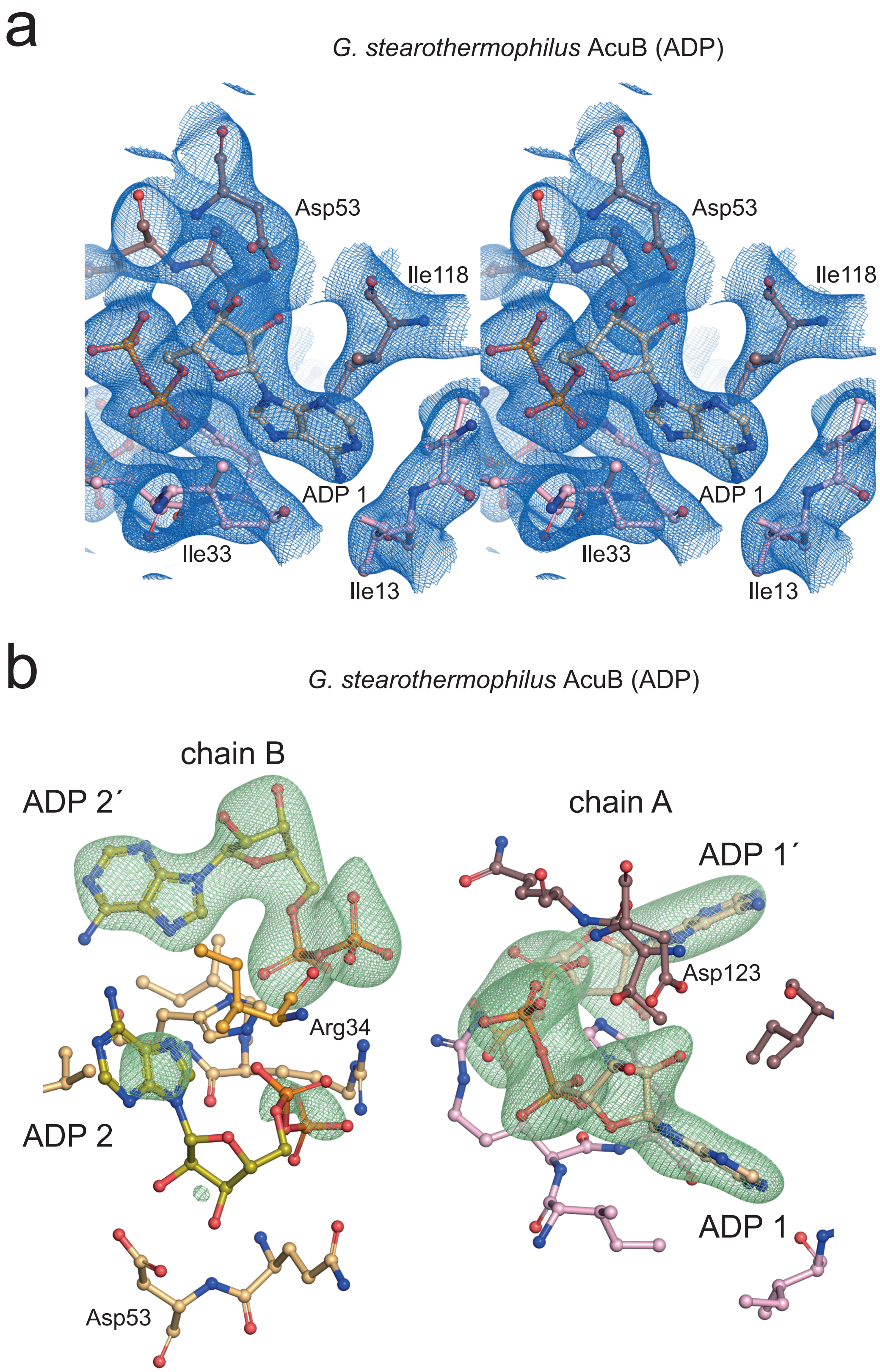


**Supplementary Fig. 24: Representative electron density obtained for the crystal structure of GsAcuB in complex with ADP (PDB: 9SAX** [https://doi.org/10.2210/pdb9SAX/pdb]**).**

**a.** Shown is a closeup of 2Fo-Fc electron density the type I and type II adenine nucleotide binding site of monomer A countered at 1σ. The figure was created with PyMOL[1].

**b.** Fo-Fc omit map countered at 3σ for adenine nucleotides bound to AcuB as indicated. The figure was created with PyMOL[1].

a

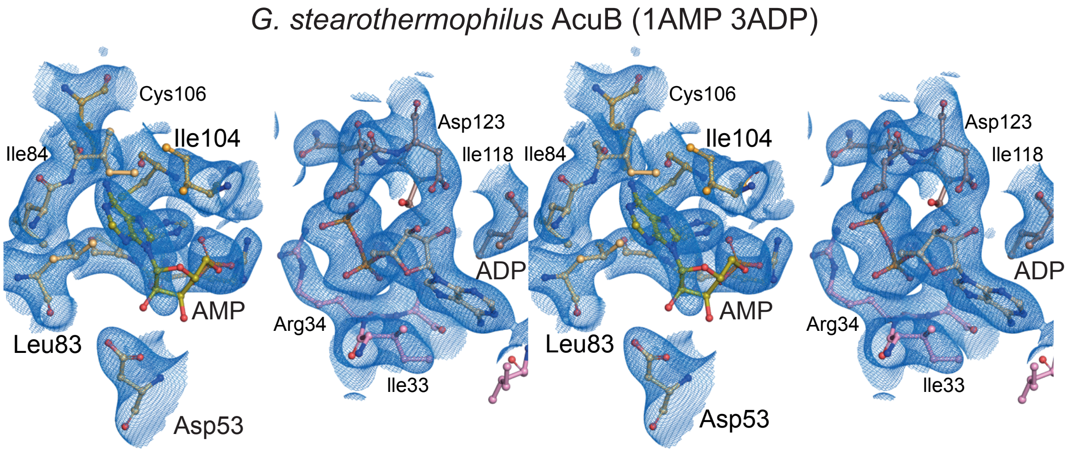


b

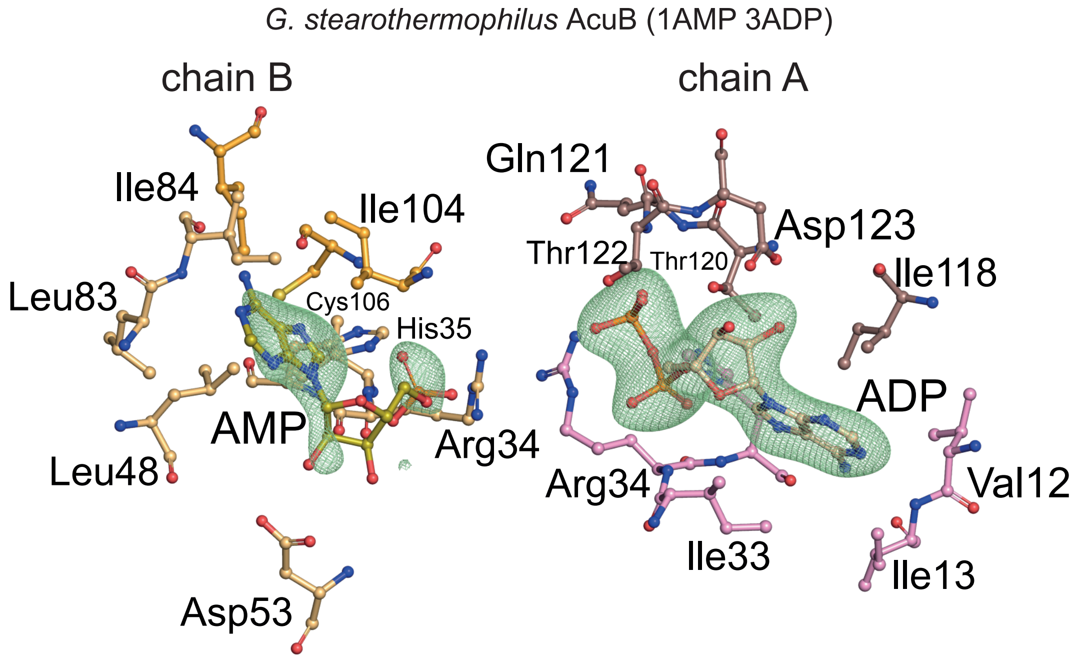


**Supplementary Fig. 25: Representative electron density obtained for the crystal structure of GsAcuB in complex with AMP and ATP (PDB: 9S4Z** [https://doi.org/10.2210/pdb9S4Z/pdb]**).**

**a.** Shown is a closeup of 2Fo-Fc electron density the type I and type II adenine nucleotide binding site of monomer A countered at 1σ. The figure was created with PyMOL[1].

**b.** Fo-Fc omit map countered at 3σ for adenine nucleotides bound to AcuB as indicated. The figure was created with PyMOL[1].

a

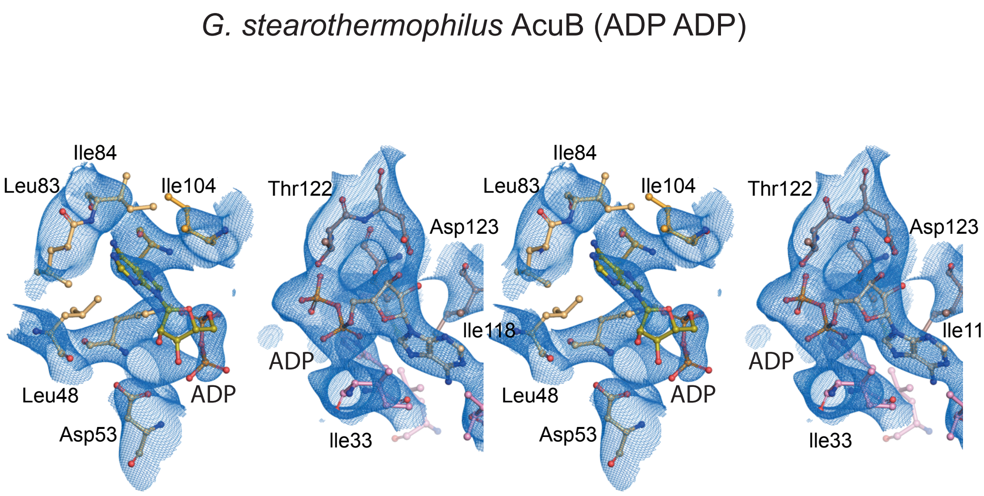


b

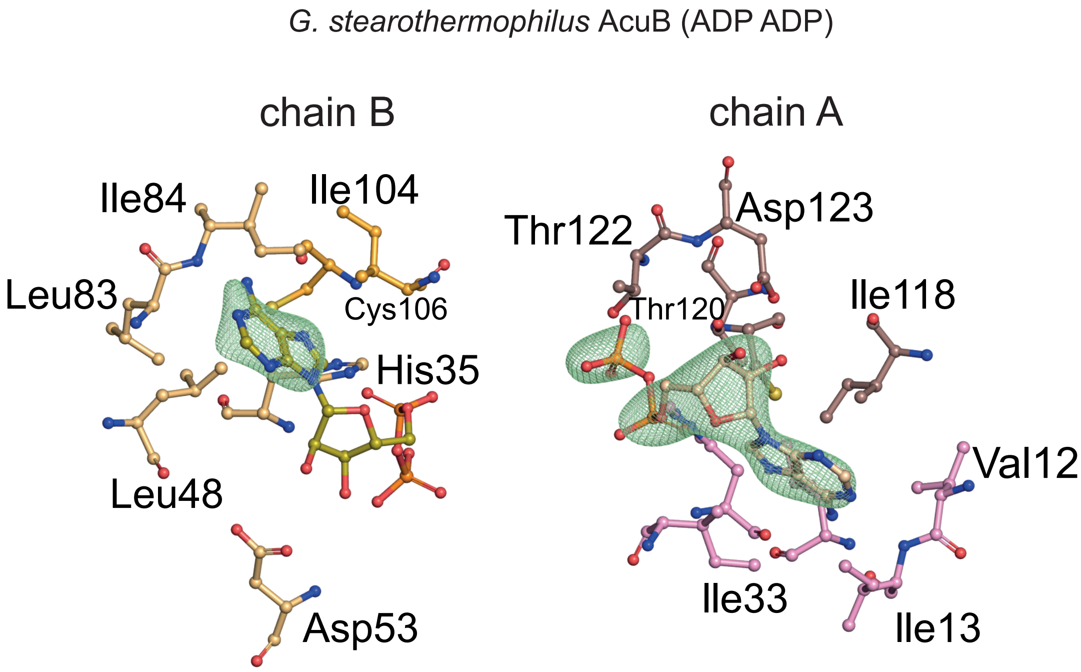


**Supplementary Fig. 26: Representative electron density obtained for the crystal structure of GsAcuB in complex with AMP and ATP (PDB: 9S50** [https://doi.org/10.2210/pdb9S50/pdb]**).**

**a.** Shown is a closeup of 2Fo-Fc electron density the type I and type II adenine nucleotide binding site of monomer A countered at 1σ. The figure was created with PyMOL[1].

**b.** Fo-Fc omit map countered at 3σ for adenine nucleotides bound to AcuB as indicated. The figure was created with PyMOL[1].

a

G. stearothermophilus AcuB (Ap4A)

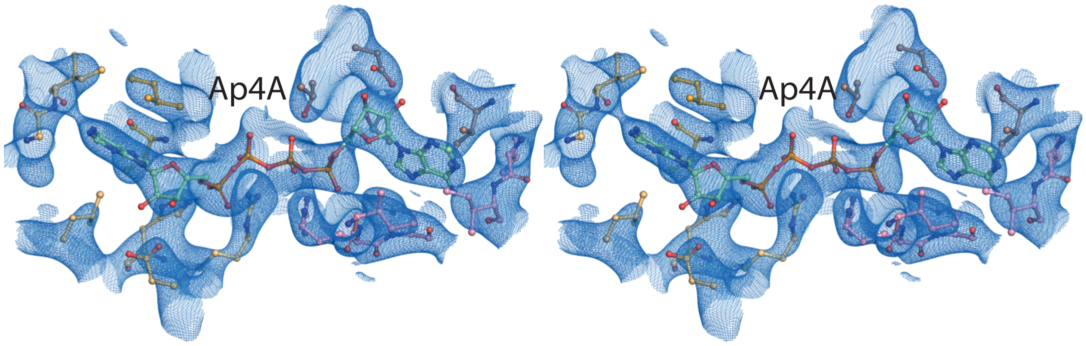


b

G. stearothermophilus AcuB (Ap4A)

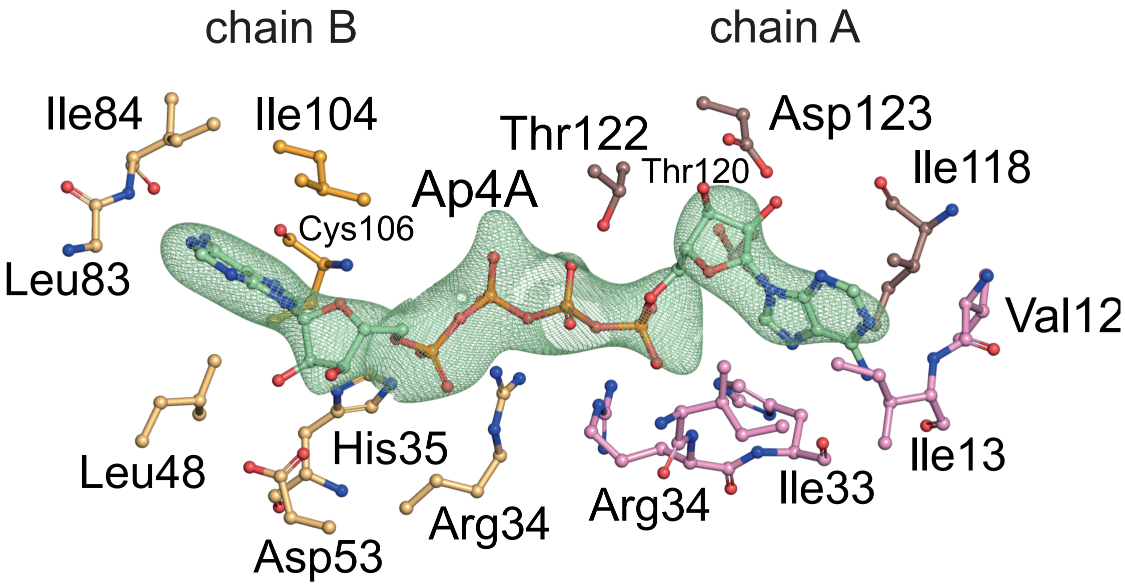


**Supplementary Fig. 27: Representative electron density obtained for the crystal structure of GsAcuB in complex with Ap4A (PDB: 9SAY [https://doi.org/10.2210/pdb9SAY/pdb]).**

**a.** Shown is a closeup of 2Fo-Fc electron density the type I and type II adenine nucleotide binding site of monomer A countered at 1σ. The figure was created with PyMOL[1].

**b.** Fo-Fc omit map countered at 3σ for Ap4A bound to AcuB as indicated. The figure was created with PyMOL[1].

a

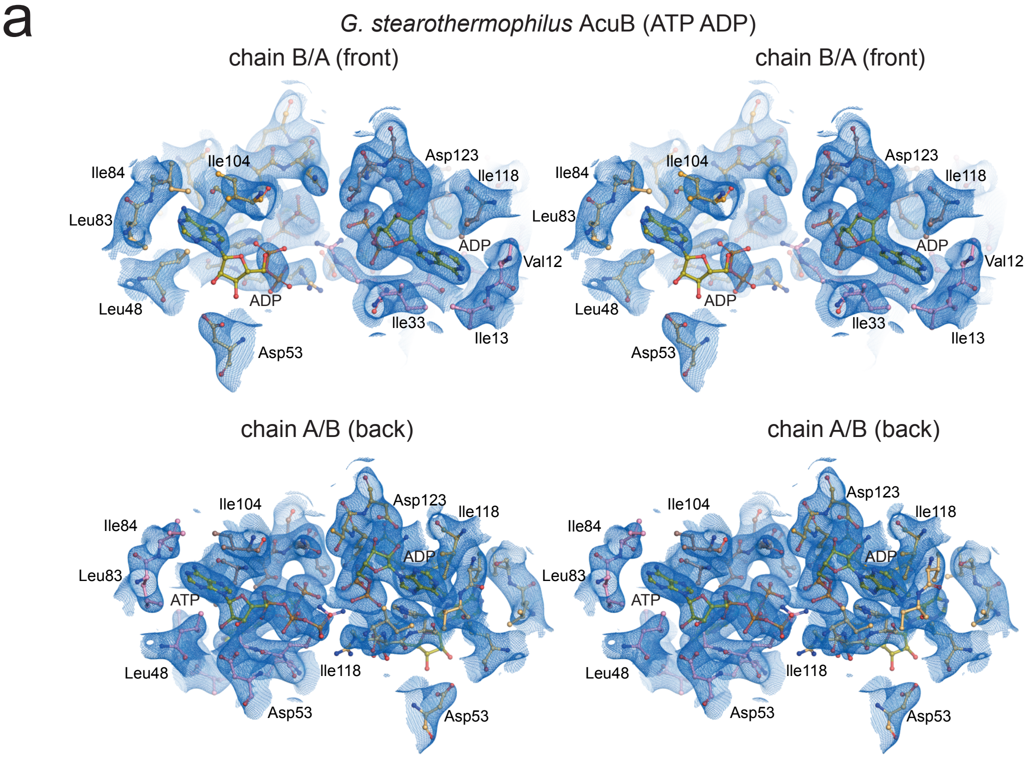


b

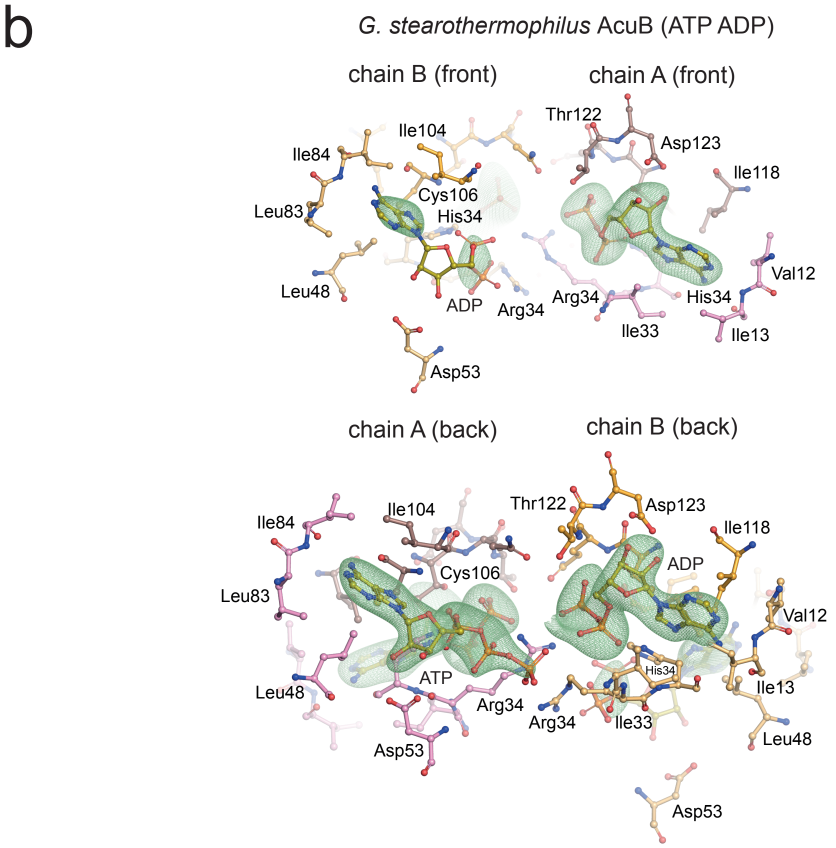


**Supplementary Fig. 28: Representative electron density obtained for the crystal structure of GsAcuB in complex with ADP and ATP (PDB: 9S51 [https://doi.org/10.2210/pdb9S51/pdb]).**

**a.** Shown is a closeup of 2Fo-Fc electron density the type I and type II adenine nucleotide binding site of monomer A countered at 1σ. The figure was created with PyMOL[1,4].

Fo-Fc omit map countered at 3σ for adenine nucleotides bound to AcuB as indicated. The figure was created with PyMOL[1].

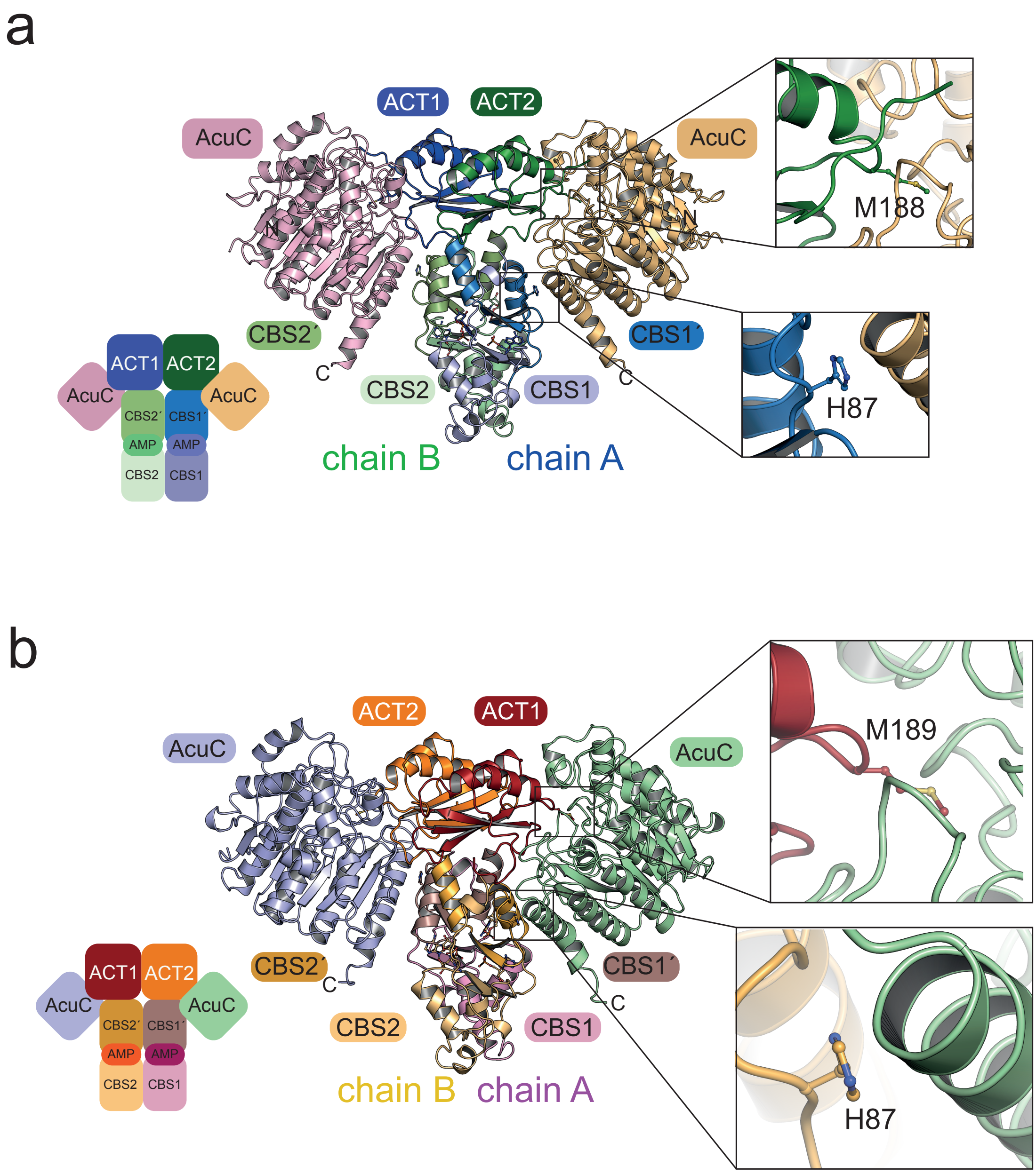


**Supplementary Fig. 29: Localization of the mutations in BsAcuB/GsAcuB selected to validate the AlphaFold3 model of GsAcuB$_2$•2GsAcuC.** M188 (*B. subtilis*) /M189 (*G. stearothermophilus*) is located in the C-terminal ACT domain and H86 (*B. subtilis*)/H87 (*G. stearothermophilus*) in the Bateman domain. Both residues are in contact distance to AcuC. M188/M189 penetrates deeply into the AcuC substrate binding channel. The AlphaFold3 model of the BsAcuB$_2$•2BsAcuC complex in complex with 4AMP is shown in a, the model of GsAcuB$_2$•2GsAcuC in complex with 4 AMP in b. Both predictions have reliable confidence scores (Supplementary Table 7). The figure was created with PyMOL[1].

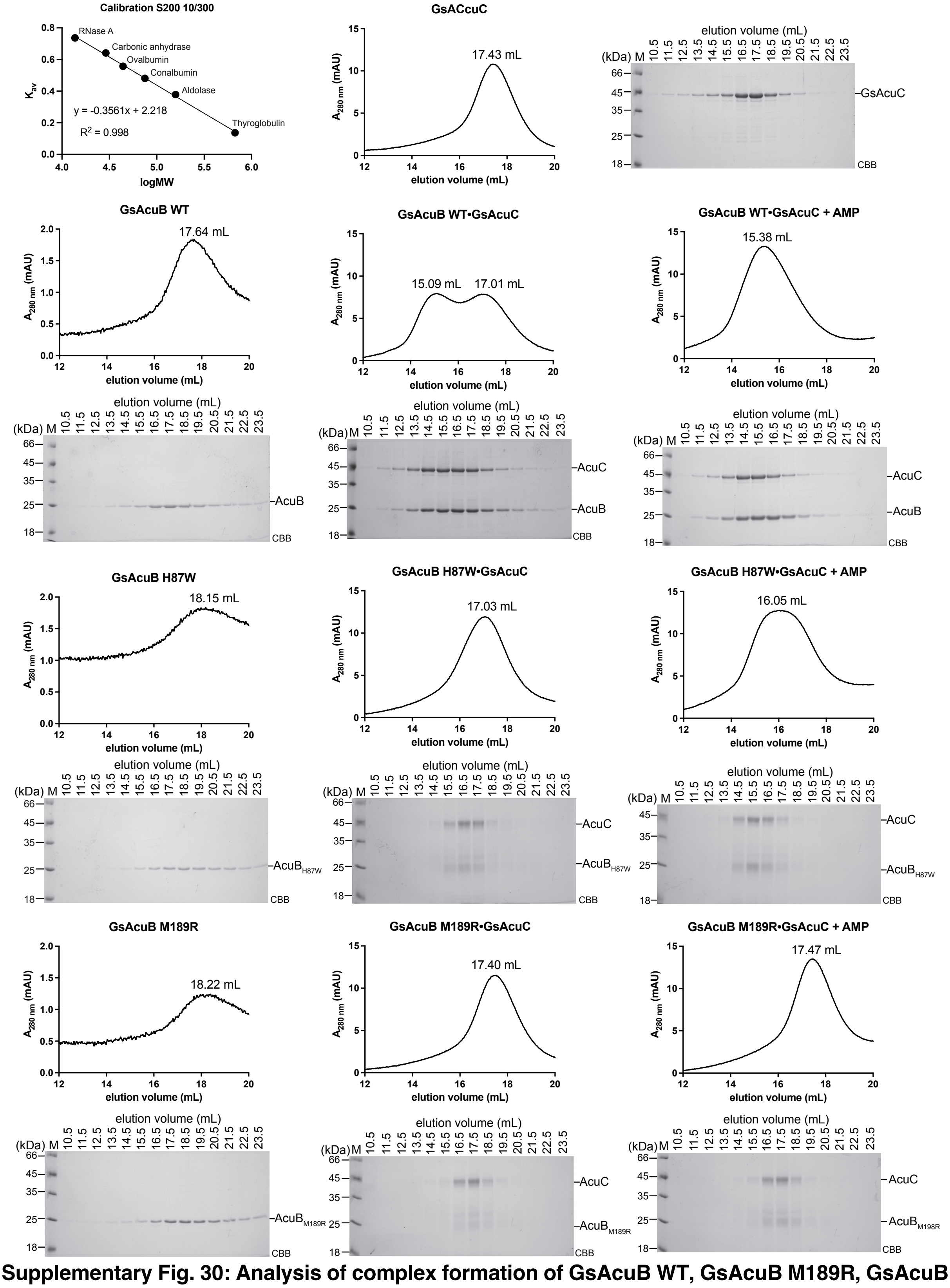


**Supplementary Fig. 30: Analysis of complex formation of GsAcuB WT, GsAcuB M189R, GsAcuB H87W and GsAcuC in dependence of presence of AMP by analytical SEC on an S200 10/300 column.** Complex formation between GsAcuC wildtype and GsAcuB is detectable, while the mutant GsAcuB M189R does not form a complex. Compared to GsAcuB wildtype for GsAcuB H87W complex formation is detectable but impaired. Elution was followed by recording the absorption at 280 nm ($A_{280\ nm}$) in milli absorbance units (mAU). The fractions of the analytical SEC were analyzed by SDS-PAGE. The lane labelled with M represents the protein molecular weight marker. The SDS-PAGE gels were stained with Coomassie-brilliant blue (CBB). Source data are provided as Source Data file.

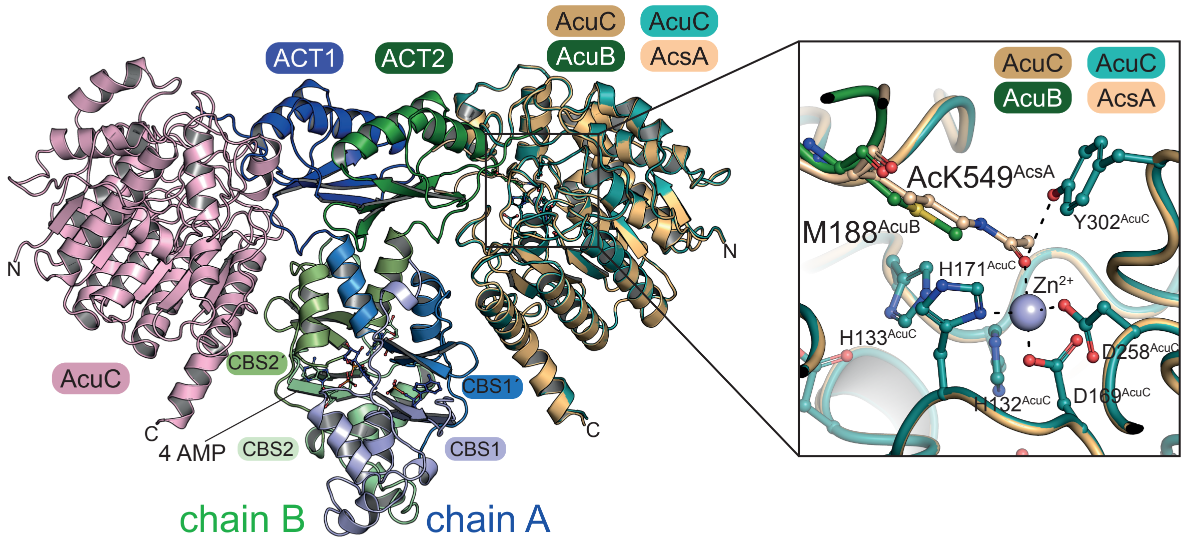


**Supplementary Fig. 31: Binding of AcuB to AcuC blocks substrate access to the AcuC active site.** AlphaFold3 model of heterotetrameric $BsAcuB_2$•2BsAcuC in complex with four molecules of AMP was superimposed with a complex of BsAcuC•$Zn^{2+}$ and a peptide derived from K549-acetylated AMP-forming acetyl-CoA synthetase (BsAcsA AcK549). Both models show reliable confidence scores (Supplementary Table 7). It is obvious that M188 of BsAcuB inserts into the substrate binding channel guiding towards the AcuC active site. The AcK549 of AcsA superimposes well with M189. Binding of AcuB to AcuC blocks the access of substrate to the active side explaining the observed inhibitory effect of AcuB. The active site $Zn^{2+}$-ion is shown as a sphere. H171, D169, D258 of AcuC and the substrate acetyl-group coordinate the $Zn^{2+}$-ion. Y302 binds the carbonyl oxygen of the substrate's acetyl group thereby orienting and polarizing it for catalysis. The figure was created with PyMOL[1].

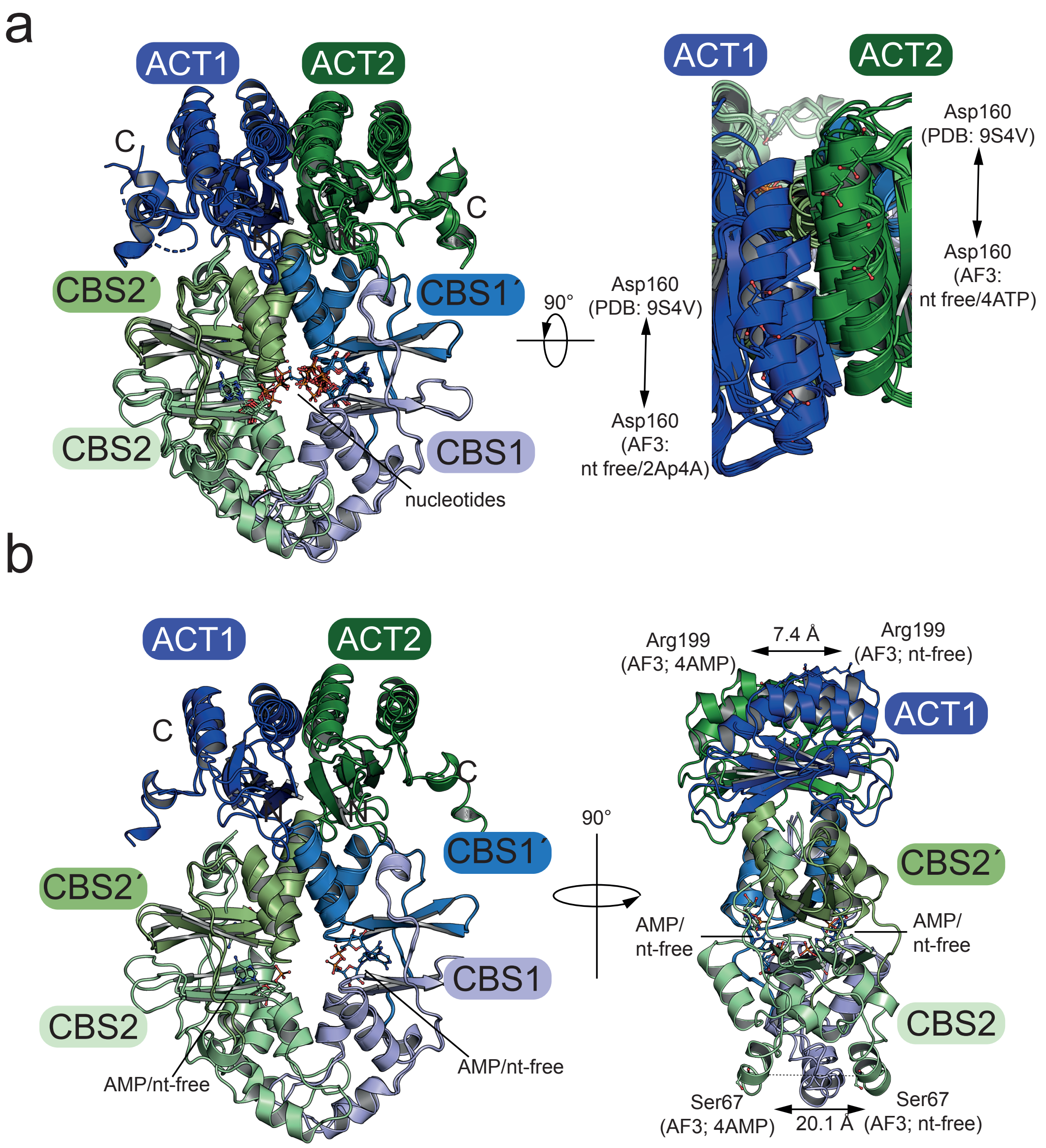


**Figure 32: Comparison of AlphaFold3 structural models suggest that BsAcuB has inherent structural flexibility dependent on the nucleotide loading state.**

**a.** AlphaFold3 models of BsAcuB in complexes with four molecules of AMP, ADP, ATP, two molecules of AMP and two molecules of ADP, or two molecules of Ap4A were structurally aligned on the experimental structure of BsAcuB determined in complex with two molecules AMP and two molecules of ADP. The alignments were done on the Bateman domain of monomer A (residues 1-131). The structures show a high structural similarity of the nucleotide-bound BsAcuB proteins (Supplementary Table 9). In the ACT domains there is some structural variability in the position of the ACT domains towards the Bateman domains. The AlphaFold3 models might not represent the real structural dynamics as it modeled two ATP-molecules of chain B in the complex of BsAcuB•4ATP with their $\gamma$-phosphates coming too close to be capable to bind in this conformation. Moreover, the model of BsAcuB in complex with two molecules of Ap4A shows that each Ap4A molecule binds to each AcuB monomer rather than bridging both monomers in the dimer as shown by the experimental structure of GsAcuB•2 Ap4A.The figure was created with PyMOL[1].

**b.** Superposition of the AlphaFold3 models of nucleotide free BsAcuB and BsAcuB•4AMP as representative for nucleotide-bound BsAcuB. The nucleotide free BsAcuB shows remarkable structural differences to the nucleotide-bound BsAcuB-proteins. Shown are conformational differences in the Bateman domain as indicated by the distance of the C$\alpha$-atoms of Ser67 of 20.1 Å and of Arg199 of 7.4 Å. This suggests that nucleotide-binding brings BsAcuB in a more rigid state as also supported by the nanoDSF data. Overall, AlphaFold3 might not be able to accurately model the dynamics in BsAcuB, which is present as shown by MD-simulations. Particularly, electrostatic effects by repulsion of negatively-charged adenine-nucleotide phosphates act over long-distances affecting the dynamics in the Bateman domain. The figure was created with PyMOL[1].

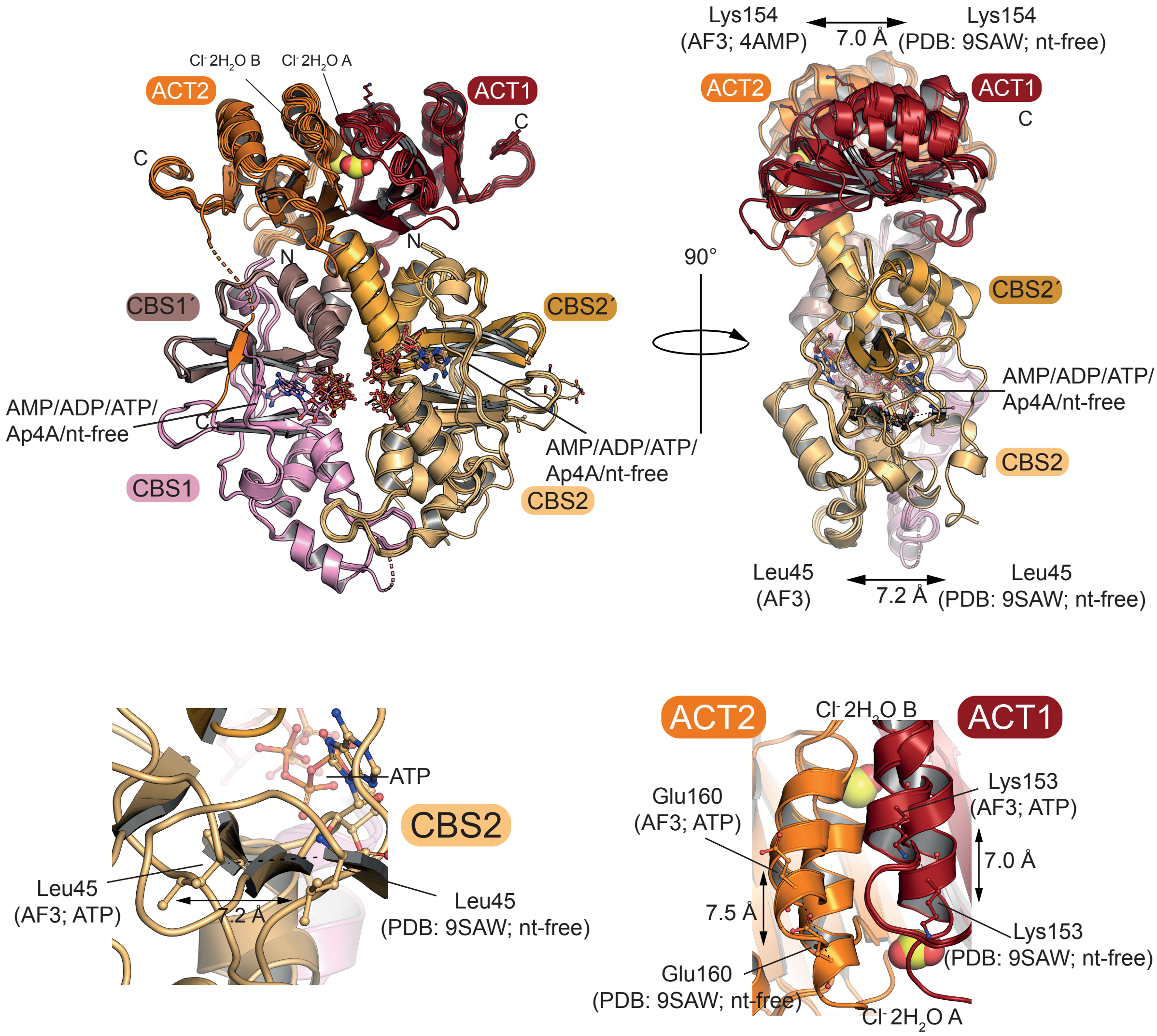


**Figure 33: Comparison of AlphaFold3 structural models suggest that GsAcuB has inherent structural flexibility dependent on the nucleotide loading state.**
AlphaFold3 models of GsAcuB in complexes with four molecules of AMP, ADP, ATP, combinations thereof (2AMP+2ADP; 2AMP+2ATP, 2ADP+2ATP, nt-free) or two molecules of Ap4A were structurally aligned on the experimental structure of GsAcuB determined in the nucleotide free, empty state (PDB: 9SAW [https://doi.org/10.2210/pdb9SAW/pdb]). The alignments were done on the Bateman domain of monomer A (residues 1-131). The structures show a high overall structural similarity of the AlphaFold3 models of the GsAcuB proteins compared (Supplementary Table 9). However, there are differences in the experimentally determined structure of nucleotide free GsAcuB towards the AlphaFold3 models in the Bateman domain and the ACT domains as indicated by showing distances of Cα-atoms of selected residues. As indicated for the BsAcuB AlphaFold3 models, the AlphaFold3 models might not represent the real conformational dynamics as it modeled two ATP-molecules of chain A and B in the complex of GsAcuB•4ATP with their γ-phosphates at a distance of 1.9 Å. The model of GsAcuB in complex with two molecules of Ap4A shows that each Ap4A molecule binds to each GsAcuB monomer rather than bridging both monomers in the dimer as shown by the experimental structure of GsAcuB•2Ap4A. Highlighted are conformational differences in the Bateman domain as indicated by the distance of the Cα-atoms of Leu45 of 7.2 Å and in the ACT domains indicated by the distance of Cα Lys153 of 7.0 Å and Glu160 of 7.5 Å. This suggests that nucleotide-binding affects the conformation of GsAcuB as also supported by the nanoDSF data. Overall, AlphaFold3 might not be able to accurately model the dynamics in GsAcuB, which is present as shown by MD-simulations. Particularly, electrostatic effects by repulsion of negatively-charged adenine-nucleotide phosphates act over long-distances affecting the dynamics in the Bateman domain. The figure was created with PyMOL[1].

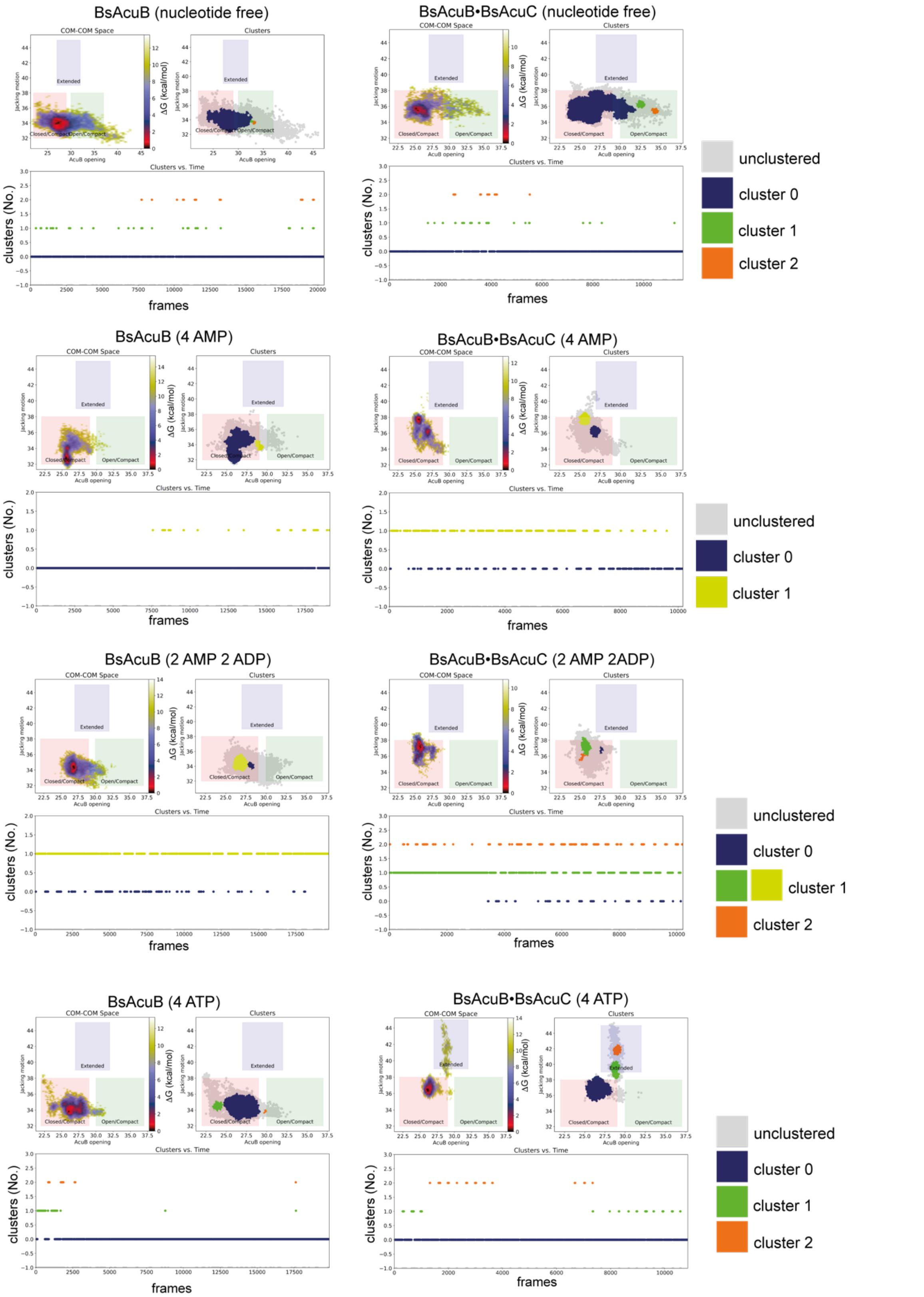


**Supplementary Fig. 34: Cluster map analyses for MD simulations of BsAcuB in the nucleotide free state and in complexes with 4AMP, 2AMP+2ADP and 4ATP.** The analyses suggests that BsAcuB and the complex of BsAcuB and BsAcuC adopts different preferred conformations depending on the nucleotide loading of BsAcuB. While nucleotide free BsAcuB shows strong conformational flexibility in the Bateman domains, binding of AMP nucleotides in both, AMP loaded and AMP/ADP loaded BsAcuB shows a decrease in this flexibility. Binding of ATP to BsAcuB results in adopting extended conformations if BsAcuB is present in complex with BsAcuC. These extended conformations cannot be observed if BsAcuB is in its uncomplexed form.

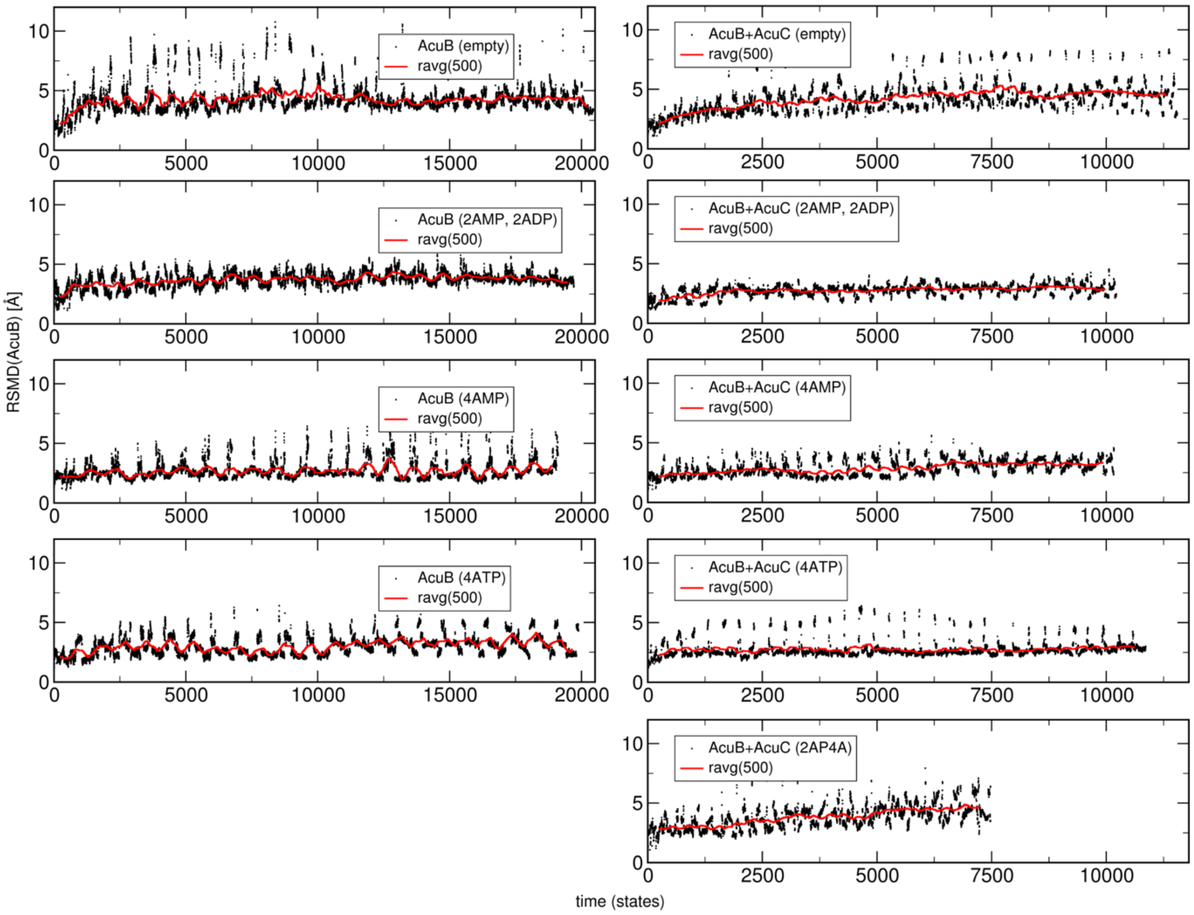


**Supplementary Fig. 35: Convergence analysis of TIGER2h-PE replica exchange simulations.** Backbone RMSD of AcuB-dimer for simulations of AcuB-dimer alone (left panels) and the AcuB+AcuC complex (right panels) plotted as a function of simulation time (states) for the indicated nucleotide loading conditions. Black dots represent instantaneous RMSD values, and the red line corresponds to a 500-frame running average. In all systems, the running averages plateau after initial equilibration and fluctuate around stable mean values without systematic drift, indicating that the trajectories repeatedly traverse the same conformational ensemble. These results support convergence and sampling within a confined structural basin, without progressive structural divergence

**Supplementary Table 1:** Analysis of the molecular weight and oligomeric state of *B. subtilis* proteins, BsAcsA, BsAcuA, BsAcuB and complexes thereof by analytical size-exclusion chromatography on a S200 10/300 column. If several peaks were observed the elution volume of the maxima is shown as they appear and these were labelled with a and b. Shown is the expected proteins/complex (exp. Protein/complex), the expected molecular weight (exp. MW) and the calculated molecular weight (calc. MW) as determined using a calibration curve.

| protein | nucleotide added | exp. protein/complex | exp. MW (Da) | elution volume (mL) | calc. MW (Da) | oligomeric state (calc. MW/exp.MW) |
|---|---|---|---|---|---|---|
| **BsAcsA** | | | 64892 | 14.54 | 129315 | 1.99 |
| **BsAcuA** | | | 25484 | 19.43 | 17872 | 0.70 |
| **BsAcuA•BsAcuB** | | BsAcuB | 25484 | 16.6 | 56180 | 2.21 |
| **BsAcuA•BsAcuB** | | BsAcuA | 25484 | 19.57 | 16888 | 0.66 |
| **BsAcuB** | | | 25406 | 17.56 | 38094 | 1.50 |
| **BsAcuC** | | | 44273 | 17.27 | 42838 | 0.97 |
| **BsAcuB•BsAcuC (a)** | | heterotrimer (2 AcuB & 1 AcuC)<br>heterotetramer (2 AcuB & 2 AcuC) | 95085<br>139358 | 15.16 | 100619 | 1.06<br>0.72 |
| **BsAcuB•BsAcuC (b)** | | BsAcuB<br>BsAcuC | 25046<br>44273 | 17.20 | 44068 | 1.74<br>1.00 |
| **BsAcuB•BsAcuC (a)** | AMP | heterotrimer (2 AcuB & 1 AcuC)<br>heterotetramer (2 AcuB & 2 AcuC) | 95085<br>139358 | 14.82 | 115461 | 1.21<br>0.83 |
| **BsAcuB•BsAcuC (b)** | AMP | BsAcuB<br>BsAcuC | 25046<br>44273 | 17.35 | 41473 | 1.63<br>0.94 |
| **BsAcuB•BsAcuC (a)** | ADP | heterotrimer (2 AcuB & 1 AcuC)<br>heterotetramer (2 AcuB & 2 AcuC) | 95085<br>139358 | 15.26 | 96628 | 1.02<br>0.69 |
| **BsAcuB•BsAcuC (b)** | ADP | BsAcuB<br>BsAcuC | 25046<br>44273 | 17.38 | 40972 | 1.61<br>0.93 |
| **BsAcuB•BsAcuC (a)** | ATP | heterotrimer (2 AcuB & 1 AcuC)<br>heterotetramer (2 AcuB & 2 AcuC) | 95085<br>139358 | 15.28 | 95849 | 1.00<br>0.69 |
| **BsAcuB•BsAcuC (b)** | ATP | BsAcuB<br>BsAcuC | 25046<br>44273 | 17.37 | 41138 | 1.62<br>0.93 |
| **BsAcuB•BsAcuC (a)** | Ap4A | heterotrimer (2 AcuB & 1 AcuC)<br>heterotetramer (2 AcuB & 2 AcuC) | 25046<br>44273 | 15.91 | 74278 | 0.78<br>0.53 |
| **BsAcuB•BsAcuC (b)** | Ap4A | BsAcuB<br>BsAcuC | 25046<br>44273 | 17.38 | 40972 | 1.61<br>0.93 |

**Supplementary Table 2:** Melting temperatures ($T_m$) obtained by nanoDSF measurements for BsAcuB, GsAcuB, BsACT in their apo forms and upon incubation with AMP, ADP, ATP and Ap4A. Each measurement was performed in triplicates for BsAcuA (*n*=3) or quadruplicates for GsAcuB and BsACT (*n*=4). Shown are the means of the $T_m$ values and the standard deviation (Stdev) and the difference of the $T_m$-values, $\Delta T_m$, of the nucleotide treated compared to untreated samples. Source Data are provided in Supplementary Data files.

| **protein** | **nucleotide** | **$T_m$ (°C) (mean±Stdev.)** | **$\Delta T_m$ (°C) (apo to nt)** |
|---|---|---|---|
| BsAcuB | - (apo state) | 44.3±0.4 | 0 |
| | AMP | 55.5±0.1 | +11.2 |
| | ADP | 59.5±0.1 | +15.2 |
| | ATP | 58.2±0.1 | +13.9 |
| | Ap4A | 73.5±0.2 | +29.2 |
| GsAcuB | - (apo state) | 66.7±0.2 | 0 |
| | AMP | 73.8±0.2 | +7.1 |
| | ADP | 78.4±0.2 | +11.7 |
| | ATP | 77.9±0.5 | +11.2 |
| | Ap4A | 83.9±0.6 | +17.2 |
| BsACT | - (apo state) | 76.1±0.3 | 0 |
| | AMP | 76.4±0.7 | +0.3 |
| | ADP | 75.7±0.5 | -0.4 |
| | ATP | 76.2±0.3 | +0.1 |
| | Ap4A | 76.2±0.2 | +0.1 |

**Supplementary Table 3:** Analysis of the molecular weight and oligomeric state of *Geobacillus stearothermophilus* proteins, GsAcuB and GsAcuC and complexes thereof by analytical size-exclusion chromatography on a S200 10/300 column. If several peaks were observed the elution volume of the maxima is shown as they appear and these were labelled with a and b. Shown is the expected proteins/complex (exp. Protein/complex), the expected molecular weight (exp. MW) and the calculated molecular weight (calc. MW) as determined using a calibration curve.

| protein | nucleotide added | exp. protein/complex | exp. MW (Da) | elution volume (mL) | calc. MW (Da) | oligomeric state (calc. MW/exp.MW) |
|---|---|---|---|---|---|---|
| **GsAcuB** | | | 24860 | 16.95 | 42989 | 1.73 |
| **GsAcuC** | | | 43430 | 17.23 | 38324 | 0.88 |
| **GsAcuB•GsAcuC (a)** | | heterotrimer (2 AcuB & 1 AcuC) | 93150 | 14.9 | 99677 | 1.07 |
| | | heterotetramer (2 AcuB & 2 AcuC) | 136580 | | | 0.73 |
| **GsAcuB•GsAcuC (b)** | | GsAcuB | 24860 | 17.2 | 38798 | 1.56 |
| | | GsAcuC | 43429 | | | 0.89 |
| **GsAcuB•GsAcuC** | AMP | heterotrimer (2 AcuB & 1 AcuC) | 93150 | 14.63 | 111353 | 1.20 |
| | | heterotetramer (2 AcuB & 2 AcuC) | 136580 | | | 0.82 |
| **GsAcuB•GsAcuC (a)** | ADP | heterotrimer (2 AcuB & 1 AcuC) | 93150 | 14.95 | 97653 | 1.05 |
| | | heterotetramer (2 AcuB & 2 AcuC) | 136580 | | | 0.72 |
| **GsAcuB•GsAcuC (b)** | ADP | GsAcuB | 24860 | 17.18 | 39118 | 1.57 |
| | | GsAcuC | 43429 | | | 0.90 |
| **GsAcuB•GsAcuC** | ATP | heterotrimer (2 AcuB & 1 AcuC) | 93150 | 15.65 | 73278 | 0.79 |
| | | heterotetramer (2 AcuB & 2 AcuC) | 136580 | | | 0.54 |
| **GsAcuB•GsAcuC** | Ap4A | GsAcuB | 24860 | 16.95 | 42989 | 1.73 |
| | | GsAcuC | 43429 | | | 0.99 |

**Supplementary Table 4: Data collection and refinement statistics for the structures of *Bacillus subtilis* and *Geobacillus stearothermophilus* AcuB.**

| | *B, subt.* AMP,ADP | *G. stear.* none | *G. stear.* AMP | *G. stear.* ADP | *G. stear.* Ap4A | *G. stear.* AMP, ATP | *G. stear.* AMP, ADP | *G. stear.* ADP, ATP |
|---|---|---|---|---|---|---|---|---|
| **Data collection** | D_1292149441 | D_129214943 | D_1292149466 | D_1292149450 | D_1292149451 | D_1292149458 | D_1292149455 | D_1292149459 |
| | DESY P13 | BESSY 14.1 | BESSY 14.1 | DESY P13 | BESSY 14.1 | DESY P13 | DESY P13 | DESY P13 |
| Space group | $P2_122_1$ | $P2_12_12_1$ | $P2_12_12_1$ | $P2_12_12_1$ | $P2_12_12_1$ | $P2_12_12_1$ | $P2_12_12_1$ | $P2_12_12_1$ |
| Cell dimensions *a*, *b*, *c* (Å) | 67.31 79.78 153.79 | 45.62 96.78 98.97 | 52.69 96.41 101.87 | 52.49 96.49 101.09 | 52.30 96.95 101.71 | 51.56 96.54 101.44 | 52.30 96.67 101.38 | 52.43 96.51 101.16 |
| Resolution (Å) | 50 – 2.64 | 50 – 2.00 | 50 – 2.30 | 50 – 2.76 | 50 – 3.35 | 50 – 3.06 | 50 – 2.62 | 50 – 2.53 |
| $R_{sym}$ | 0.137 (0.145) | 0.109 (1.176) | 0.276 (1.549) | 0.128 (0.527) | 0.433 (1.570) | 0.227 (1.610) | 0.071 (0.702) | 0.170 (1.085) |
| Unique reflections | 24477 (3021) | 30440 (2219) | 23740 (5002) | 26470 (6233) | 7877 (1393) | 10462 (2027) | 15706 (2151) | 17740 (3230) |
| $I / \sigma\sigma(I)$ | 10.0 (1.1) | 20.7 (2.8) | 11.7 (2.0) | 12.8 (2.8) | 8.0 (2.0) | 8.3 (1.3) | 21.0 (2.22) | 12.4 (13.1) |
| Completeness (%) | 97.4 (99.2) | 100.0 (100.0) | 100.0 (100.0) | 99.5 (99.3) | 100.0 (100.0) | 99.6 (99.9) | 97.5 (84.6) | 100.0 (100.0) |
| Redundancy | 10.0 (10.3) | 13.1 (12.8) | 12.9 (13.6) | 6.9 (6.8) | 12.7 (12.6) | 12.8 (13.9) | 12.7 (8.6) | 13.6 (2.4) |
| Wilson B (Å$^2$) | 73.6 | 35.7 | 41.4 | 63.7 | 61.2 | 84.0 | 72.7 | 67.4 |
| **Refinement** | | | | | | | | |
| Resolution (Å) | 2.64 | 2.00 | 2.30 | 2.76 | 3.35 | 3.06 | 2.62 | 2.53 |
| No. reflections | 23200 | 28917 | | | | | | |
| $R_{work}$ / $R_{free}$ | 0.212 / 0.273 | 0.190 / 0.245 | 0.200 / 0.252 | 0.183 / 0.249 | 0.229 / 0.262 | 0.223 / 0.287 | 0.181 / 0.236 | 0.192 / 0.243 |
| No. atoms (non-H) | | | | | | | | |
| Protein | 5103 | 3309 | 3187 | 3147 | 3154 | 3144 | 3099 | 3138 |
| Ligand/ion | 150 | 16 | 108 | 124 | 108 | 121 | 120 | 125 |
| Water | 14 | 0 | 55 | 10 | 0 | 6 | 17 | 4 |
| *B*-factors | | | | | | | | |
| Protein (mc/sc) | 74.6 (71.8/76.2) | 32.2 (29.3/33.7) | 35.3 (33.2/36.3) | 70.8 (68.0/71.4) | 76.6 /75.3/77.3) | 67.0 (64.9/68.1) | 64.9 (62.1/66.3) | 68.5 (66.5/69.7) |
| Ligand/ion | 67.8 | 48.5 | 37.3 | 58.0 | 73.1 | 111.5 | 77.8 | 65.3 |
| Water | 65.9 | 39.4 | 36.4 | 44.4 | - | 57.1 | 56.7 | 25.8 |
| R.m.s. dev (Molprobity) | | | | | | | | |
| Bond lengths (Å) | 0.010 | 0.011 | 0.011 | 0.022 | 0.008 | 0.009 | 0.011 | 0.010 |
| Bond angles (°) | 1.60 | 1.95 | 1.93 | 2.15 | 1.58 | 1.91 | 1.99 | 2.08 |
| Ramachandran (Molprobity) favored/outlier | 97.0/0.5 | 98.7/0.5 | 98.0/0.3 | 97.7/0.3 | 90.1/0.0 | 97.2/0.3 | 98.7/0.0 | 97.2/0.0 |

[1*]/[2*]: for each structure one crystal was used.

[3*]: values in parentheses are for highest-resolution shell.

**Supplementary Table 5: B-factors and occupancy values obtained for the adenine nucleotides bound to type I and type II sites in monomers A-C in BsAcuB or monomers A,B in GsAcuB.** The B-factors so not contain TLS-contribution.

| Protein | Type I site | Type II site |
|---|---|---|
| BsAcuB/GsAcuB | B-factor (Å$^2$)/occupancy (%) (nucleotide) | |
| BsAcuB AMP ADP (PDB: 9S4V) (2 AMP + 2ADP) | | |
| Monomer A | 53/100 (ADP) | 57/100 (AMP) |
| Monomer B | 74/100 (ADP) | 63/100 (AMP) |
| Monomer C | 80/100 (ADP) | 82/100 (AMP) |
| GsAcuB AMP (PDB: 9S52) (4 AMP) | | |
| Monomer A | 28/100 (AMP) | 35/100 (AMP) |
| Monomer B | 53/100 (AMP) | 54/100 (AMP) |
| GsAcuB ADP (PDB: 9S4X) (4 ADP) | | |
| Monomer A | 51/100 (ADP) | 59/100 (ADP) |
| Monomer B | 59/100 (ADP) | 83/50 (ADP) |
| GsAcuB AMP ATP (PDB: 9S4Z) (3 ADP + 1AMP) | | |
| Monomer A | 63/100 (ADP) | 64/100 (ADP) |
| Monomer B | 59/100 (ADP) | 145/100 (AMP) |
| GsAcuB AMP ATP (PDB: 9S50) (TM1) (4 ADP) | | |
| Monomer A | 87/100 (ADP) | 125/100 (ADP) |
| Monomer B | 117/70 (ADP) | 143/70 (AMP) |
| GsAcuB ADP ATP (PDB: 9S51) (TD1) (3 ADP + 1 ATP) | | |
| Monomer A | 51/100 (ADP) | 64/80 (ATP) |
| Monomer B | 53/70 (ADP) | 100/50 (ADP) |
| GsAcuB Ap4A (PDB: 9SAY) (2 Ap4A) | | |
| Monomer A | 103/100 (Ap4A part 1) | 91/100 (Ap4A part 2) |
| Monomer B | 86/100 (Ap4A part 1) | 65/100 (Ap4A part 2) |
| GsAcuB empty (PDB: 9SAW) (no nucleotide) | | |
| Monomer A | empty | empty |
| Monomer B | empty | empty |

**Supplementary Table 6:** Analysis of the molecular weight and oligomeric state of the isolated *B. subtilis* ACT domain of BsAcuB. Moreover, a complex formation of BsACT with the deacetylases AcuC from *B. subtilis* (BsAcuC), *B. anthracis* (BaAcuC) and *Geobacillus stearothermophilus* (GsAcuC) was analysed. The construct BsAcuB 132-214 corresponds to the C-terminal ACT domain, BsACT. The analyses were conducted on an analytical size-exclusion chromatography S200 10/300 column. Shown is the expected proteins/complex (exp. Protein/complex), the expected molecular weight (exp. MW) and the calculated molecular weight (calc. MW) as determined using a calibration curve.

| protein | nucleotide added | exp. protein/complex | exp. MW (Da) | elution volume (mL) | calc. MW (Da) | oligomeric state (calc. MW/exp.MW) |
|---|---|---|---|---|---|---|
| **BsAcuB 132-214 (BsACT)** | | | 10510 | 18.9 | 25959 | 2.47 |
| **BsAcuC** | | | 44273 | 17.43 | 46451 | 1.05 |
| **BaAcuC** | | | 44823 | 17.92 | 38261 | 0.85 |
| **GsAcuC** | | | 43430 | 17.82 | 39806 | 0.92 |
| **BsAcuB 132-214 (BsACT)**<br>**BsAcuC** | | heterotrimer (2 AcuB & 1 AcuC)<br>heterotetramer (2 AcuB & 2 AcuC) | 65293<br>109566 | 17.52 | 44825 | 0.69<br>0.41 |
| **BsAcuB 132-214 (BsACT)**<br>**BsAcuC** | | BsAcuB 132-214<br>BsAcuC | 10510<br>44273 | 17.52 | 44825 | 4.27<br>1.01 |
| **BsAcuB 132-214 (BsACT)**<br>**BaAcuC** | | heterotrimer (2 AcuB & 1 AcuC)<br>heterotetramer (2 AcuB & 2 AcuC) | 65843<br>110666 | 17.71 | 41578 | 0.63<br>0.38 |
| **BsAcuB 132-214 (BsACT)**<br>**BsAcuC** | | BsAcuB 132-214<br>BaAcuC | 10510<br>44823 | 17.71 | 41578 | 3.96<br>0.93 |
| **BsAcuB 132-214**<br>**GsAcuC** | | heterotrimer (2 AcuB & 1 AcuC)<br>heterotetramer (2 AcuB & 2 AcuC) | 64450<br>107880 | 17.63 | 42916 | 0.67<br>0.40 |
| **BsAcuB 132-214 (BsACT)**<br>**BsAcuC** | | BsAcuB 132-214<br>BaAcuC | 10510<br>43430 | 17.63 | 42916 | 4.08<br>0.99 |

**Supplementary Table 7: AlphaFold3 quality indicators pTM+ipTM for the predictions shown in this study.**

| Protein/Protein complex | pTM+ipTM | ranking score |
|---|---|---|
| $BsAcuB_2$•4AMP•2BsAcuC | 0.86+0.86 | 0.86 |
| $BsAcuB_2$•2AMP•2ADP•2BsAcuC | 0.85+0.85 | 0.85 |
| BsAcuC•$Zn^{2+}$-$AcsA_{544-554}$ AcK549 (Protenix Server)[5] | 0.95+0.88 | 0.91 |
| BsAcuC•$Zn^{2+}$ | 0.95+0.96 | 0.96 |
| BsAcuC | 0.97 | |
| BsAcuB nucleotide free | 0.70+0.69 | 0.69 |
| BsAcuB•4AMP | 0.82+0.79 | 0.81 |
| BsAcuB•4ADP | 0.80+0.77 | 0.79 |
| BsAcuB•4ATP | 0.80+0.77 | 0.78 |
| BsAcuB•2Ap4A | 0.77+0.75 | 0.75 |
| BsAcuB•2AMP•2ADP | 0.81+0.79 | 0.81 |
| BsAcuB•2AMP•2ATP | 0.80+0.77 | 0.79 |
| BsAcuB•2ADP•2ATP | 0.80+0.77 | 0.79 |
| $GsAcuB_2$•4AMP•2GsAcuC | 0.80+0.77 | 0.79 |
| GsAcuB nucleotide free | 0.80+0.78 | 0.79 |
| GsAcuB•4AMP | 0.83+0.80 | 0.81 |
| GsAcuB•4ADP | 0.81+0.78 | 0.79 |
| GsAcuB•4ATP | 0.81+0.77 | 0.78 |
| GsAcuB•2Ap4A | 0.79+0.76 | 0.76 |
| GsAcuB•2AMP•2ADP | 0.83+0.80 | 0.81 |
| GsAcuB•2AMP•2ATP | 0.83+0.81 | 0.82 |
| GsAcuB•2ADP•2ATP | 0.81+0.78 | 0.78 |

**Supplementary Table 8:** Analysis of the molecular weight and oligomeric state of *Geobacillus stearothermophilus* AcuB, GsAcuB, mutants thereof and analysis of complex formation of these GsAcuB proteins with GsAcuC and complexes thereof by analytical size-exclusion chromatography on a S200 10/300 column. The experiments were performed in presence and absence of AMP. If several peaks were observed the elution volume of the maxima is shown as they appear and these were labelled with a and b. Shown is the expected proteins/complex (exp. Protein/complex), the expected molecular weight (exp. MW) and the calculated molecular weight (calc. MW) as determined using a calibration curve.

| protein | AMP | exp. protein/complex | exp. MW (Da) | elution volume (mL) | calc. MW (Da) | oligomeric state (calc. MW/exp.MW) |
|---|---|---|---|---|---|---|
| **GsAcuB** | - | GsAcuB• GsAcuB | 24861 | 17,64 | 47854 | 1,92 |
| **GsAcuB H87W** | - | GsAcuB• GsAcuB | 24839 | 18,15 | 39092 | 1,57 |
| **GsAcuB M189R** | - | GsAcuB• GsAcuB | 24815 | 18,22 | 38022 | 1,53 |
| **GsAcuC** | - | GsAcuC | 43430 | 17,43 | 52010 | 1,20 |
| **GsAcuB•GsAcuC (a)** | - | heterotrimer (2 AcuB & 1 AcuC) | 93151 | 15.09 | 131541 | 1.41 |
| | | heterotetramer (2 AcuB & 2 AcuC) | 136580 | | | 0,96 |
| **GsAcuB•GsAcuC (b)** | - | AcuB | 24861 | 17,01 | 61435 | 2.47 |
| | | AcuC | 43430 | | | 1.41 |
| **GsAcuB•GsAcuC (a)** | AMP | heterotrimer (2 AcuB & 1 AcuC) | 93151 | 15.38 | 117252 | 1.26 |
| | | heterotetramer (2 AcuB & 2 AcuC) | 136581 | | | 0.86 |
| **GsAcuB H87W•GsAcuC (a)** | - | heterotrimer (2 AcuB & 1 AcuC) | 93107 | 17.03 | 60949 | 0.65 |
| | | heterotetramer (2 AcuB & 2 AcuC) | 136537 | | | 0.45 |
| **GsAcuB H87W•GsAcuC (b)** | - | AcuB | 24839 | 17.03 | 60949 | 2.45 |
| | | AcuC | 43430 | | | 1.40 |
| **GsAcuB H87W•GsAcuC (a)** | AMP | heterotrimer (2 AcuB & 1 AcuC) | 93107 | 16.05 | 89895 | 0.07 |
| | | heterotetramer (2 AcuB & 2 AcuC) | 136537 | | | 0.66 |
| **GsAcuB H87W•GsAcuC (b)** | AMP | AcuB | 24839 | 16.05 | 89895 | 3.62 |
| | | AcuC | 43430 | | | 2.07 |
| **GsAcuB M189R•GsAcuC (a)** | - | heterotrimer (2 AcuB & 1 AcuC) | 93059 | 17.40 | 52632 | 0.57 |
| | | heterotetramer (2 AcuB & 2 AcuC) | 136489 | | | 0.39 |
| **GsAcuB M189R•GsAcuC (b)** | - | AcuB | 24815 | 17.40 | 52632 | 2.12 |
| | | AcuC | 43430 | | | 1.21 |
| **GsAcuB M189R•GsAcuC (a)** | AMP | heterotrimer (2 AcuB & 1 AcuC) | 93059 | 17.47 | 51191 | 0.55 |
| | | heterotetramer (2 AcuB & 2 AcuC) | 136489 | | | 0.38 |
| **GsAcuB M189R•GsAcuC (b)** | AMP | AcuB | 24815 | 17.47 | 51191 | 2.06 |
| | | AcuC | 43430 | | | 1.18 |

**Supplementary Table 9:** Structural similarity of the experimental BsAcuB AMP ADP (PDB: 9SAV) structure and nucleotide free GsAcuB towards AlphaFold3 predicted structures of BsAcuB and GsAcuB in complexes with various adenine nucleotides.

| Protein/Protein complex | RMSD (Å) |
|---|---|
| **Similarity towards experimental structure of BsAcuB AMP ADP (PDB: 9S4V) (2 AMP + 2ADP)** | |
| BsAcuB nucleotide free | 0.67 |
| BsAcuB•4AMP | 0.52 |
| BsAcuB•4ADP | 0.53 |
| BsAcuB•4ATP | 0.49 |
| BsAcuB•2Ap4A | 0.57 |
| BsAcuB•2AMP•2ADP | 0.50 |
| BsAcuB•2AMP•2ATP | 0.50 |
| BsAcuB•2ADP•2ATP | 0.49 |
| **Similarity towards experimental structure of nucleotide free GsAcuB (PDB: 9SAW)** | |
| GsAcuB nucleotide free | 0.83 |
| GsAcuB•4AMP | 0.88 |
| GsAcuB•4ADP | 0.83 |
| GsAcuB•4ATP | 0.82 |
| GsAcuB•2Ap4A | 0.82 |
| GsAcuB•2AMP•2ADP | 0.86 |
| GsAcuB•2AMP•2ATP | 0.86 |
| GsAcuB•2ADP•2ATP | 0.82 |

**Table 10: Overview of simulated systems, denoting system content, total simulation time across all replicas including quenching phases and total number of cycles, average temperature changes during replica swaps for TIGER2h-PE algorithm and average swap probability. The number of cycles is also the number of structures generated.**

| Run | System content | T (µs) (cycles) | ΔT/X (K) | P(X) (%) |
|---|---|---|---|---|
| AcuB | AcuB, no nucleotides | 3.9 (20447) | 14.5 | 31.2 |
| AcuB 2AMP 2ADP | AcuB, 2xAMP, 2xADP | 3.8 (19711) | 12.5 | 28.2 |
| AcuB 4AMP | AcuB, 4xAMP | 3.7 (19110) | 12.9 | 28.3 |
| AcuB 4ATP | AcuB, 4xATP | 3.8 (19885) | 9.1 | 22.3 |
| AcuB•AcuC | AcuB•AcuC, no nucleotides | 2.2 (11547) | 11.1 | 25.7 |
| AcuB•AcuC 2AMP 2ADP | AcuB•AcuC, 2xAMP, 2xADP | 2.0 (10363) | 8.0 | 20.5 |
| AcuB•AcuC 4AMP | AcuB•AcuC, 4xAMP | 2.0 (10309) | 9.9 | 23.9 |
| AcuB•AcuC 4ATP | AcuB•AcuC, 4xATP | 2.1 (10858) | 4.5 | 13.8 |
| AcuB•AcuC Ap4A | AcuB•AcuC, 4xAP4A | 1.4 (7475) | 11.4 | 26.3 |

**References:**


1. The PyMOL Molecular Graphics System, Version 3.0, Schrödinger, LLC.
2. Madeira, F. et al. The EMBL-EBI Job Dispatcher sequence analysis tools framework in 2024. *Nucleic Acids Res* **52**, W521-W525 (2024).
3. Robert, X. & Gouet, P. Deciphering key features in protein structures with the new ENDscript server. *Nucleic Acids Res* **42**, W320-4 (2014).
4. Jurrus, E. et al. Improvements to the APBS biomolecular solvation software suite. *Protein Sci* **27**, 112-128 (2018).
5. ByteDance AML AI4Science Team, X.C., Yuxuan Zhang, Chan Lu, Wenzhi Ma, Jiaqi Guan, Chengyue Gong, Jincai Yang, Hanyu Zhang, Ke Zhang, Shenghao Wu, Kuangqi Zhou, Yanping Yang, Zhenyu Liu, Lan Wang, Bo Shi, Shaochen Shi, Wenzhi Xiao. Protenix - Advancing Structure Prediction Through a Comprehensive AlphaFold3 Reproduction. *bioRxiv 2025.01.08.631967* (2025).
