# Supplementary material for: AcuB senses cellular energy charge to coordinate acetyl-CoA synthesis in bacteria
Source: Nat Commun. 2026 Apr 24;17:3815. doi: 10.1038/s41467-026-71006-w (PMC13121692; doi:10.1038/s41467-026-71006-w)
Supplement: Supplementary file 2 — Description of Additional Supplementary File [file 41467_2026_71006_MOESM2_ESM.docx]

**Description of additional Supplementary Data Files:**

**Supplementary Data 1:** NanoDSF data obtained for BsAcuB in the nucleotide free state and upon addition of AMP, ADP, ATP and Ap4A.

**Supplementary Data 2:** NanoDSF data obtained for GsAcuB in the nucleotide free state and upon addition of AMP, ADP, ATP and Ap4A.

**Supplementary Data 3:** NanoDSF data obtained for BsACT domain in the nucleotide free state and upon addition of AMP, ADP, ATP and Ap4A.

**References:**

1. ByteDance AML AI4Science Team, X.C., Yuxuan Zhang, Chan Lu, Wenzhi Ma, Jiaqi Guan, Chengyue Gong, Jincai Yang, Hanyu Zhang, Ke Zhang, Shenghao Wu, Kuangqi Zhou, Yanping Yang, Zhenyu Liu, Lan Wang, Bo Shi, Shaochen Shi, Wenzhi Xiao. Protenix - Advancing Structure Prediction Through a Comprehensive AlphaFold3 Reproduction. *bioRxiv 2025.01.08.631967* (2025).
